# Ethics and action pathways for psychologists in a time of climate crisis

**DOI:** 10.3389/fpsyg.2026.1770041

**Published:** 2026-05-21

**Authors:** Carolyn Houlding, B. Mackenzie Barnett, Sarah Colton, Elizabeth Houlding, Fred Schmidt, Barna Konkolý Thege

**Affiliations:** 1 Waypoint Research Institute, Waypoint Centre for Mental Health Care, Penetanguishene, ON, Canada; 2 Department of Psychology, Lakehead University, Thunder Bay, ON, Canada; 3 Ontario Institute for Studies in Education, University of Toronto, Toronto, ON, Canada; 4 Children's Hospital of Eastern Ontario, Ottawa, ON, Canada; 5 Children's Centre Thunder Bay, Thunder Bay, ON, Canada; 6 Department of Psychiatry, University of Toronto, Toronto, ON, Canada

**Keywords:** climate crisis, professional ethics in psychology, activism, advocacy, collective action

## Abstract

The climate crisis is a public and mental health emergency and amplified risk for those who are already vulnerable with significant implications for population-level quality of life and human rights. The relevance and urgency of the climate crisis is reflected in numerous “calls to action” from psychologists, health professionals, scientists, academics, and others to prioritize professional engagement with this issue. Such engagement is highly consistent with professional ethical codes and psychologists are uniquely positioned to contribute to mitigation and adaptation efforts due to their content expertise, professional training, research practice and networks affording them positions of influence. The present paper highlights three broad issues. First, it positions psychologists' involvement as an ethical and professional responsibility. The ethics codes and position papers of a number of psychology organizations are reviewed. Second, the paper discusses potential contributions of psychological science to collective and individual action as well as expertise in different areas of practice (clinical, educational, health, organizational, community, conservation, research). Last, practical ways in which any psychologist (student or qualified) in various roles (e.g., clinician, researcher, employee), in different arenas (work, community), utilizing different strategies (e.g., individual and collective advocacy/action) are identified. The paper emphasizes the importance of collective action to enact systems change. We argue that across their numerous roles and subdisciplines, psychologists not just can but should support necessary mitigation and adaptation efforts, empowering themselves and colleagues to mobilize and address the most pressing and impactful societal issue of our time.

## Introduction

“Tipping points” are approaching beyond which the destructive impacts of climate change will be rapid and irreversible (McCowan, 2020). The UN Secretary-General António Guterres commented that “Our world needs climate action on all fronts—everything, everywhere, all at once.” (Harris, 2023, para 4). Further, the societal consequences of climate change (some of which are already “locked in”) will substantially impact the funding context necessary to deliver public (including mental health) services.

Climate change has been declared a global health emergency and health crisis (Abassi et al., 2023; Lee and Harber, 2025; Løkken Nordrum et al., 2025). A host of adverse physical (e.g., Faerron Guzmán et al., 2025; Rocque et al., 2021; Romanello et al., 2021; Solomon and LaRocque, 2019), mental and cognitive health outcomes (e.g., Arnout, 2023; Cleland et al., 2022; Crane et al., 2022; Hayes et al., 2019; Lowe et al., 2018; Mahendran et al., 2021; Singh et al., 2022; Van Susteren, 2018) directly and indirectly associated with climate change are already occurring and are well-documented. Impacts of climate and environmental damage can be through exposure to extreme weather, wildfires, water- and air pollution, food insecurity and the spread of infectious diseases (Romanello et al., 2022). Direct and measurable mental health and cognitive consequences include increased aggression, anxiety, depression, suicidal behavior as well as neurogenerative disorders (Van Susteren, 2018). Further, Van Susteren (2018) comments about indirect impacts that include, “sweeping psychological effects… from the massive-scale disruptions that are the guts of the psychological toll and show why immediate action is needed and why alarm is so legitimate.” (p. 25). This includes “hundreds of millions” of climate refugees resulting from drought, famine and conflict over access to resources (including fossil fuels).

Climate change amplifies inequities and is inextricably linked to a host of related social justice concerns (Baird, 2008; Henritze et al., 2023; UNICEF, 2015). This includes racism, colonization, poverty, sexism with disproportionate impact on children and elders (Greer, 2023; UNICEF, 2015).

The severity and urgency of the climate crisis is reflected in statements by prominent health and mental health organizations. The World Health Organisation (WHO) (United Nations, 2025) identified the climate crisis as a health crisis and in a Lancet editorial, Atwoli et al. (2021) articulate that “The greatest threat to global public health is the continued failure of world leaders to keep the global temperature rise below 1.5 °C and to restore nature.” (p. 941). The UK's (Royal College of Psychiatrists 2021) declared a climate and ecological emergency and other medical associations have recognized the imperative to act (e.g., Canadian Medical Association, n.d.).

Within medicine, professional contributions to climate stewardship have been situated in an ethical context (e.g., Auckland et al., 2022; Howard et al., 2023; Timmermann et al., 2026; Yassaie and Brooks, 2025). For instance, Timmermann et al. (2026) recently published a review of physicians' ethical responsibilities in relation to the climate, which were often framed around Beauchamp and Childress's (2019) Principles of Biomedical Ethics and language related to Duty of Care. There have also been numerous “calls to action” for engagement with the climate crisis for other health professionals (e.g., Brennan and Madden, 2023; Lilienfeld et al., 2018; MacPherson and Wynia, 2017; Maibach et al., 2021; Power et al., 2022; Solomon and LaRocque, 2019).

Importantly, there have also been “calls to action” to psychologists or psychological scientists (Aron, 2019; Association of Child Psychotherapists, 2021; Galway and Field, 2023; Gimalova and Milton, 2019; O'Hare, 2022; Van Susteren, 2018) and other mental health workers (Li et al., 2022; Power et al., 2022). These include reflections on the potential contributions of psychology and the enormity of the implications. For instance, the Climate Minds Coalition (2024) in the UK generated a consensus statement and open letter articulating their position and vision for the role of psychology in addressing the crisis.

Professional concern also extends to scientists and academics. There have been numerous articles calling on scientists and academics within and outside of climate sciences to become more actively engaged in nontraditional ways: that is, to actively contribute beyond conducting research and reporting scientific findings related to climate and environmental breakdown (Dupont et al., 2025; Fossen, 2025; Gardner et al., 2021; Green, 2020; Perrin et al., 2025; Racimo et al., 2022; Rossa-Roccor et al., 2021; Urai and Kelly, 2023; Wyatt et al., 2024). Thus, it is evident that there is an established and growing concern among health professionals and scientists. Further, there is a movement toward explicitly situating this concern utilizing professional ethical lens, notably thus far, principally, among physicians.

Arguably, within the context of climate breakdown, psychologists bear unique professional and ethical responsibility to act (Van Susteren, 2018; Whitmarsh, 2020). This is because climate change can be understood as an issue essentially reflecting human behavior and mindset in their relationship to the environment (Adams, 2021; Clayton, 2024; Pearson et al., 2016). The UK Association of Clinical Psychologists (Morgan et al., 2020) issued a statement emphasizing use of clinical skills in conceptualizing, modeling engagement, supporting and encouraging others to engage. They argue, “we must connect with the despair and work through our denial, supporting others to do so too, including those with power, because we must act.” The unique skills and expertise of psychology as a discipline can and should be marshaled to address this immense challenge.

Despite this widespread concern among health professionals and academics and endorsement by prominent health organizations including psychologists, the response of the health/academic community to these calls has been limited (cf. Gardner et al., 2021; Green, 2020; Power et al., 2022; Wyatt et al., 2024). This may reflect numbers of personal and professional barriers to engagement with this issue (cf. Dupont et al., 2025; Gifford, 2011, 2021; Latter et al., 2024). Psychologists do not always recognize the issue as directly pertinent to their role (McCunn et al., 2025). This may be due to limited understanding of their potential mandate or role if their research or practice roles are not directly relevant to climate change.

Therefore, it appears that there is a need to strengthen awareness and engagement of psychologists in their professional capacities. This includes in their understanding of the relevance of their ethics codes to involvement as well as their understanding of potential contributions. To this end, the present paper will: (a) review the ethics codes and position papers of numerous psychology associations, (b) reference systemic barriers to effective actions, (c) consider psychological science that is relevant to climate change mitigation, and (d) offer practical suggestions for what psychologists can do in the many roles they occupy. The paper aims to be relevant to all members within the discipline of psychology at any level of qualification and within any subdiscipline or areas of practice. For parsimony, we use the term “psychologist[s]” throughout to denote this entire group.

## Guiding documents for professional psychology: ethics codes and position papers

To understand the nature and degree to which psychology as a profession has already started to engage with the climate crisis, we examined ethics codes and position papers from an international sample of psychological associations. This included the Canadian Psychological Association (CPA), the Aotearoa/New Zealand Psychological Society (NZPsS), the American Psychological Association (APA), the European Federation of Psychologists' Associations (EFPA), The British Psychological Society (BPS), and the Australian Psychological Society (APS). These professional/regulatory associations were chosen based on their attention to the climate crisis, publication of position papers, and prominence on an international level on this issue. This selection strategy has clear limitations (e.g., non-English speaking professional associations were not accessed). We chose to highlight these countries/regions, given their particular responsibility due to their colonial legacies and disproportionate contributions to carbon outputs (Kenner, 2019).

### Ethics codes

The Canadian Code of Ethics for Psychologists 4th Ed (Canadian Psychological Association, 2017) has a clear emphasis on broader societal ethical responsibilities for psychology outlined in Principle IV (“Responsibility to Society”) of the code. While it may seem to some practitioners that climate stewardship is marginal to the psychology profession (c.f. McCunn et al., 2025), within one of the most relevant standards (Ethical Standard IV.26), climate stewardship is specifically mentioned as an example for “minimal behavioral expectations” rather than an aspirational goal in this code of ethics. The same standard IV. 26 explicitly names psychologists' responsibility to adhere to international treaties, including those that aim to protect the environment. One such treaty is the 2015 Paris Agreement, which requires legally binding, country-level climate action plans, the success of which is dependent on both individual and collective action in each signing country. Therefore, our interpretation is that active climate stewardship is a clear ethical responsibility of psychologists according to the current Canadian code of ethics.

The NZPsS's ethics code (New Zealand Psychological Society, 2002) also specifies psychologists' role and responsibilities toward the broader society in which psychologists live. Specifically, Principle IV (Social Justice and Responsibility to Society) articulates the need for psychologists to consider the current problems of the societies in which they practice, speak out if societal structures are not supportive of the other principles of the code (e.g., promotion of human wellbeing), and the prohibition of psychologists engaging in (research) activities that contribute to the destruction of the environment.

In terms of the American Psychological Association's (APA) ethics code, Principle B (Fidelity and Responsibility) acknowledges that psychologists have “professional and scientific responsibilities to society and to the specific communities in which they work” (American Psychological Association, 2017). When considered alongside Principle A (Beneficence and Nonmaleficence), these provisions may be interpreted as offering a broad, though implicit, ethical basis for addressing large-scale societal harms such as those associated with climate change.

Similarly, the APS Code of Ethics (Australian Psychological Society, 2007) does not explicitly reference environmental concerns, but its principle of Propriety emphasizes psychologists' responsibility to safeguard the welfare of not just their direct clients but that of the general public as well, suggesting an orientation toward broader societal wellbeing.

The BPS Code of Ethics and Conduct (British Psychological Society, 2021)—in addition to emphasizing responsibility to the public—encourages psychologists to consider the broader impacts of their actions, demanding “respect for the welfare of humans, non-humans and the living world.” This language extends ethical concern beyond human-centered interactions and suggests a more expansive understanding of responsibility that can reasonably encompass environmental and ecological issues.

Taken together, this brief review suggests that climate stewardship is codified explicitly as a professional ethical obligation in the Canadian and Aotearoa/New Zealand code of professional ethics. In addition, some of the foundational principles present across most reviewed ethical codes (i.e., societal responsibility, justice, or the prevention of harm) offer a basis for its inclusion within the ethical scope of the profession. The variability across codes highlights that such an interpretation is more readily supported in frameworks that explicitly extend ethical responsibility beyond the individual clients to society as a whole.

### Position papers

A position paper of the Canadian Psychological Association (2021) emphasizes the role of psychological science in supporting three key strategies to address climate change. This includes climate communication to the public; supporting governmental climate policy; and promoting government research funding highlighting the relationship between health, wellbeing, and human behavior (related to the climate crisis). There is minimal attention given to how individual psychologists can support climate action as professionals outside of activities related to education, consultation, and knowledge dissemination.

The Climate Psychology Taskforce (CPTF) of the NZPsS focuses on understanding human behavior, knowledge-based interventions to stimulate public engagement, promoting community/lifestyle changes and the development and dissemination of climate-relevant educational resources (Dixon et al., 2022). The CPTF highlights the urgent need for action from psychology as a profession and from individual psychologists (Dixon et al., 2022; New Zealand Psychological Society, 2002).

In 2022, based on a history of promoting the role of psychology in addressing climate change, the APA released a position paper on climate action (APA Task Force on Climate Change, 2022). The APA Task Force highlighted six broad recommendations, which included specific strategies related to research, practice, education, advocacy, communication, and APA's energy/sustainability practices as an organization. These strategies focused on changing governmental and societal activities to address climate change, and the need for psychologists to take initiatives within and outside of professional roles.

The Australian Psychological Society's (APS) position paper (Australian Psychological Society, 2020) highlights the need to understand the psychological dimensions of the climate crisis, the contribution of human action to climate change, individual and societal level perceptions of climate risks and the need to support pro-environmental campaigns at an individual and societal level. The APS position paper states that psychology as a profession and APS “have a social and moral responsibility to play an active and leading role in climate change mitigation and adaptation” (p. 15) and that psychologists have a responsibility to use their skills in service of climate advocacy, education, and training (i.e., Recommendation 9.4).

The EFPA recently generated a particularly detailed position paper (Kałwak et al., 2024), synthesizing available research on climate action and outlined the 10 most important issues on climate change. These included ethical considerations and identified the manner in which existing knowledge can be used within the academic discipline, field of practice, support for policy change, and/or to shape the actions and attitudes of psychologists as individual private citizens.

The British Psychological Society (BPS) has a recent position paper outlining a “call to action” to psychologists (British Psychological Society, 2023). Its focus included “building confidence” in individuals, communities, and organizations to tackle the climate crisis; becoming “champions” to share policy on climate science; promoting education and communication about climate issues; and cultivating a deeper understanding of the links between the climate crisis, human rights, and inequality. This latter strategic goal is highlighted by embedding climate action within a social justice framework.

### Reflection on both types of professional guiding documents

National and international psychology associations differ significantly in the degree to which they overtly frame psychologists' involvement in climate mitigation as an *ethical imperative*. Professional ethical responsibilities related to climate stewardship can be understood from diverse ethical frameworks. This includes, for example, utilitarian consequentialism, virtue ethics, or social-contractual perspectives, which may each offer a rationale. However, the professional ethics codes and guiding documents reviewed above tend to converge on shared principles: notably beneficence/nonmaleficence (protecting human welfare and preventing harm) and justice (fairness/equity). Normative ethical theories (e.g., virtue ethics, deontology, natural law theory, consequentialism, or social contract theory) can be consulted for more detail of theory-oriented philosophical sources (Darwell, 2002; Driver, 2012; Finnis, 2011; Hursthouse, 2017; Jos, 2006).

A useful distinction relevant for the present review is between formal ethical obligations, as articulated in professional codes of ethics, and broader professional expectations, as reflected in position statements and other guiding documents of the profession. These two sources are not interchangeable but rather provide complementary insight into how the profession conceptualizes its responsibilities. While in the latter type of documents there is growing recognition that the climate crisis constitutes an important domain for psychological expertise and engagement, this recognition is less or not at all pronounced in codes of ethics, the primary source in which core ethical duties are defined for psychologists. In other words, climate-related engagement is frequently presented as something psychologists *may* contribute to, rather than something they *ought* to consider as integral to their roles. Considering the urgency and dramatic consequences of climate change, we propose that this positioning is no longer sufficient.

This non-exhaustive review of professional guiding documents outlines broad avenues for contribution and includes the ethical basis for climate action by psychologists. Psychologists' ethical codes directly or indirectly acknowledge responsibility to broader societal and justice issues, which is clearly relevant for the environmental crisis. Further, among psychology codes reviewed, Canada's and New Zealand's explicitly frame involvement of psychologists as a societal ethical issue. Hence it appears that engaging with climate adaptation or mitigation as a professional psychologist is highly consistent with ethical and professional practice. Introducing a stronger ethical mandate and/or operationalizing the existing one are actionable recommendations for all psychology organizations.

## Action for psychologists

There is widespread concern among health professionals and scientists regarding the consequences of the climate crisis, and (for some) a commitment to respond in their professional capacity (e.g., Hantel et al., 2024; Power et al., 2022). There are two broad and interrelated contributions for psychology as a discipline. First, the findings from *psychological science and behavior change* as a way to conceptualize and inform components of impactful action. Second, the contributions of psychological professionals in their *content expertise*. Each of these will be addressed below.

### Psychological science and behavior change

Psychological science, particularly within social psychology, has substantial relevance to understanding and facilitating individual behavior change, as well as collective action, movement building and maintenance. Zhao et al. (2024) highlight the importance of attending to operant factors that encourage environmentally protective behaviors through simultaneously rewarding pro-environmental behavior, while extinguishing environmentally harmful behaviors. This suggests that professionally-relevant mitigation efforts should be “low-barrier” and rewarding. For instance, through making relevant training and educational materials readily available, highlighting low-effort but impactful actions available to a broad range of psychologists, making policy/systems changes that make pro-environmental behaviors the default (e.g., by including in accreditation standards, university curricula and assignments, awards, funding, policies) and recognizing climate justice advocacy/training/work as part of psychologists' professional responsibilities. Consideration should also be given to empirical evidence regarding effective action (e.g., Gulliver et al., 2021, 2022, 2023; Ozden and Glover, 2022; Thomas-Walters et al., 2025; Whitmarsh et al., 2020), and the relative importance of different sources of emissions and hence priorities for change (Capstick et al., 2020; Ivanova et al., 2020; Monsell et al., 2021; Whitmarsh et al., 2020).

Weintrobe (2021) developed evidence-informed psychodynamic conceptualization of the widespread failure to engage in activism. This model dissects the specific psychological mechanisms (e.g., distress avoidance) underlying ignorance and denial associated with lack of engagement with the climate crisis. It is also suggested that expressing difficult emotions in relation to the climate crisis contributes to more active engagement with climate stewardship (Pigott et al., 2024). The “Living with the Climate Crisis” initiative was developed to address this: within this intervention, structured group meetings are held to help people process emotions, build resilience, and facilitate engagement with collective action on climate change.

Other barriers to taking an active role in addressing the climate crisis have also been explored. These include time constraints, lack of knowledge of appropriate action, reduced sense of urgency, concerns about professional censure or perceived need for neutrality (Finnerty et al., 2024), “social loafing” (i.e., delegating responsibility for action to others), rationalizing (believing that one is doing more than others) and believing that the consequences of inaction are not personally relevant (Dablander et al., 2024; Dupont et al., 2025; Gifford, 2011, 2021; Whitmarsh et al., 2020). Strategies to address these barriers include accessing education regarding the causes, urgency and impacts of the climate crisis and reasons for lack of progress, choosing impactful actions that are less time consuming, dialogue with colleagues regarding what meaningful action might look in a given context and discourse regarding “impartiality”—which favors the status quo, and hence injustice (Bhopal, 2023; Knight, 2020).

The ABIASCA framework maps effective activism (Gulliver et al., 2021). This framework also includes consideration of the importance of different audiences of activism (bystanders, supporters, opponents, third parties and self) and the tasks and outcomes that are relevant for each of these different audiences (Gulliver et al., 2021). The ABIASCA model also emphasizes the important task of sustaining action over time, including preventing and addressing burnout and addressing compassion fatigue in activists (Gulliver et al., 2021). The community and constructive action associated with participating in climate activism has been associated with decreased stress and isolation as well as increased sense of empowerment and agency (Bingley et al., 2022; Clayton and Parnes, 2025; Conner et al., 2023; Kleres and Wettergren, 2017; Ojala, 2012). Further, conflict with those outside the group (e.g., police) can strengthen commitment to activist roles (Vestergren et al., 2018).

Psychologists have expertise in generating, accessing and disseminating findings relevant to framing, communication and persuasion to recruit to movements (c.f. Ballew et al., 2025; Li and Su, 2018; Pelletier and Sharp, 2008; van der Linden et al., 2015). van der Linden et al.'s (2015) review of effective messaging emphasizes the clear communication of the proximity of climate change (present, local, and personal risk), including emotional and experiential elements, as well as gains from immediate action and long-term goals.

Psychologists also have expertise in the important issue of misinformation and disinformation, which is also relevant for attempting to engage and win the support of bystanders (and others). Narayan et al. (2026) described the importance of combatting disinformation generated by the fossil fuel industry as a “public health imperative.” A recent review article identified strategies for addressing misinformation including, for instance, tailoring messages for target audiences or identifying co-benefits of mitigation efforts (Mendy et al., 2024).

Climate involvement can also be associated with burnout and compassion fatigue (Conner et al., 2023; Lord et al., 2025). Psychologists are not immune to these impacts, although they may be more skilled in noticing, preventing and/or addressing them than the general public. This includes being aware of factors predicting risk of burnout and strategies to encourage resilience. Individuals are at increased risk of burnout when they are highly invested in group goals, have a strong sense of being responsible for these goals but are pessimistic about whether the goals can be achieved (Gulliver et al., 2023). Strategies to support environmental activists include nurturing social and other ties with climate organizations, participating in (at least some) action with short-term wins (Gulliver et al., 2023), focusing on what one can control, being realistic about change and finally, integrating work, activism, and life balance by shaping careers (Driscoll, 2019). Mental health interventions such as mindfulness and self-care, spending time in nature and mentoring (Driscoll, 2019; Godden et al., 2022; Gorski, 2015) are also protective.

Systems change often reflects organizing and using strategies which engage and mobilize people, sustain movements and build power (c.f. Engler and Engler, 2016; Ganz, 2010; McAlevey, 2016). For instance, recent gains by progressive political candidates successfully utilized “relational organizing” using a “snowflake” structure (Ganz, 2010; Saleem, 2026).

Thus far, our attention has focused on a limited areas of relevant psychological science (with more extensive reviews available through other documents; e.g., APA 2022). Attention will now shift to consider how psychologists should apply their knowledge, training and skills to engage with mitigation efforts in the range of roles in which they occupy.

### Psychology content expertise

**Figure 1:**
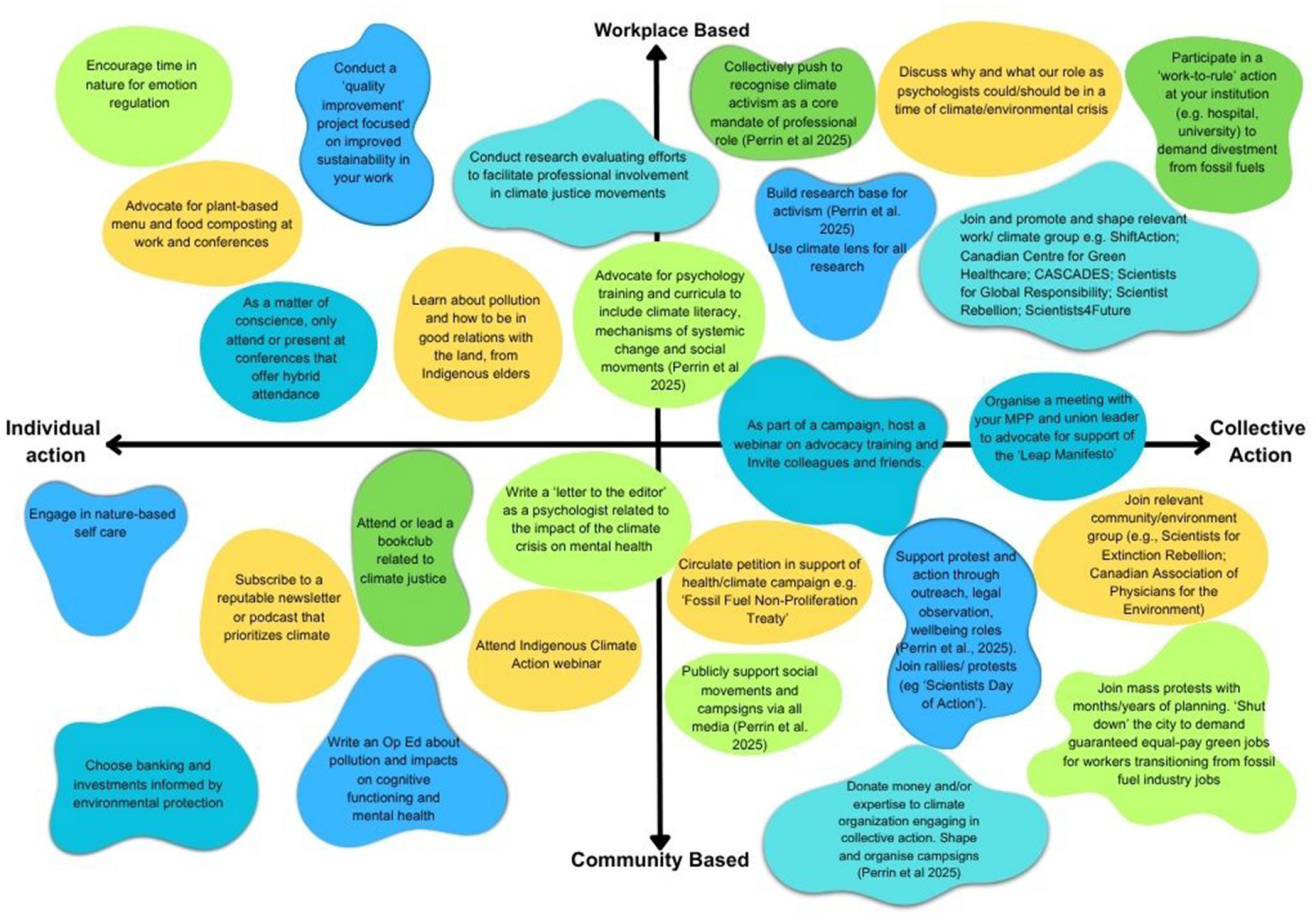
Examples of action for psychologists according to context and strategy. Examples of actions on two dimensions: employment vs. community-based and individual vs. collective strategy. Judgment regarding the position of specific examples is subjective and some actions can be executed either individually or collectively. An attempt was made to include actions with some evidence for impact (e.g., Capstick et al., 2020; Ivanova et al., 2020) and examples requiring a range of time/resources. Image adapted from (Dupont et al. 2025) with the authors' permission.

Addressing the climate emergency is highly relevant to core practices, focus and expertise for psychologists from multiple subdisciplines (for example, Table 1, Figure 1). Climate action within major psychology subdisciplines will now be reviewed.

**Table 1:** Example actions/advocacy for psychologists.

Role	Examples
All	Bring a “climate lens” to all professional activities. Develop a working knowledge of key issues (e.g., causes of climate and biodiversity crises; seriousness and urgency of climate and biodiversity crises; reasons for lack of progress; effective engagement, mobilizing and activism; optimal science communication, importance of solidarity and coalitions across movements including labor, climate)
	Engage in self-care (e.g., focused on “nature connectedness”), seek community in activism.
	Engage in self-reflection regarding relationship with nature and the climate crisis
15.6-8,-114243ptClinical counseling	Encourage “nature connection” in clients. Provide clinical support to climate activists. In the context of acceptance and commitment therapy (ACT), explore conservation volunteering (e.g., invasive species removal, tree planting). (Where appropriate) encourage nonpharmacological interventions (e.g., exercise, sleep hygiene, emotional regulation) for symptoms. Advocate for “greening” office space programming areas and program practices. Become competent in treatment of eco distress; provide training for disaster “first response”.
Health practice	
Educational	Understand and become competent in treating eco distress in youth. Support youth in nature connection, conservation and appropriate activism efforts. Support schools in implementing training for educators in addressing eco distress.
Industrial/ organizational	Support organizations to implement and maintain sustainability practices, e.g., decreased food waste and single use plastics, plant-rich menus, reusable materials. Act as content experts for effectiveness and quality management in environmental organizations. Facilitate “communities of practice” for sustainability within organizations.
Researcher	Prioritize research supporting engagement, mobilizing, collective action, climate mitigation and/or pro environmental adaptation in area of specialty. Include “climate/environmental impact” as a consideration for ethical clearance of projects. Endorse, advocate for and model sustainable practices as a scientist (e.g., avoid air travel; promote plant-rich options, minimal waste and unnecessary “merch” at conferences). Publicly and actively stand in solidarity with colleagues (national and international) to advocate for freedom of inquiry in science, including promoting social and climate justice and attending to the influence of the fossil fuel industry in academia. Write/sign “open letters” from reputable organizations as a researcher/academic (and encourage colleagues to do so).
Academic educator/trainer	Advocate for inclusion of curricula/seminars/topics related to history of the environmental movement, environmental racism and relationship between climate and other social justice topics (e.g., Indigenous communities; housing). Advocate for widespread availability of courses related to effective mobilizing, collective action, advocacy and activism. Include assignments related to participating in advocacy (e.g., meetings with elected officials, organizational decision makers) and other relevant actions.
Worker/ organization member	Speak with colleagues about meaningful, impactful action as professionals and as a collective. Learn about solidarity and coalitions across movements (e.g., labor; climate), models of collective action and strategies for “building power.” Join and contribute to psychology/climate groups (e.g., Canadian Coalition for Green Health Care; SHIFT Action for Pension Wealth and Pension Health; Scientists for Global Responsibility; Climate Minds Coalition). Build support to advocate for sustainable consumption, procurement, food and food waste and other operational practice within your organization (including fiduciary and climate responsibility in banking, investments and pensions), utilizing toolkits from existing organizations (e.g., Canadian Coalition for Green Health Care). Include pro-environmental articles in organizational newsletters, as agenda items on standing committees and as a “lens” when completing any work-based surveys. Take part in collective bargaining. Attend protests/actions as a union member. Use Earth Day and other environmentally relevant events to increase awareness and engagement of colleagues in pro environmental collective action. Advocate for plant-rich menu at work and conferences, as a default. Conduct a “quality improvement” project focused on improving sustainability in your work and in collaboration with climate organizations.
Community-based individual advocacy (as a psychologist)	Donate to climate/health organizations. Comment/share social media posts of reputable climate organizations. Write to elected officials (school trustee; municipal, provincial, federal) as a professional. Attend or lead a book club related to climate justice. Attend an Indigenous Climate Action webinar. Subscribe to reputable newsletters or podcasts that prioritize climate/environment. Write a “letter to the editor” in a local magazine as a psychologist highlighting the impact of the climate crisis on mental health.
Community-based collective action	Donate time/skills to climate/health organizations, particularly focused on tasks that are impactful and that you enjoy/are good at/bring you community. Join relevant health/science/climate organization (e.g., CAPE; Scientists for Extinction Rebellion). As part of a CAPE campaign, host a webinar and circulate a petition supporting a ban on fossil fuel advertising. As part of a campaign, be a psychology-relevant content expert for media (e.g., on disinformation, cognitive impact of pollution). As part of a campaign—participate in deputations, meet with elected officials, participate in “phone zaps,” sign and share reputable climate-relevant petitions. Join a “Scientists' Day of Action” march protesting suppression of science. Host a webinar and invite colleagues. Donate money or skills to climate organizations engaging in collective action. Engage in pro-bono support for climate activists' post-arrest for non-violent protest.

#### Clinical and counseling practice

In any clinical role (e.g., clinical, counseling), practitioners can indirectly impact emissions or biodiversity loss through therapeutic interventions; for example, through incorporation of nature-focused activities in therapy and encouragement of “nature connection” (which improves mental health and may increase commitment to pro-environmental behaviors; e.g., Coventry et al., 2021; Whitburn et al., 2019; Yeon et al., 2021 and Table 1). A related role for clinical practitioners might be to actively promote psychosocial interventions (including exercise, sleep hygiene or emotional regulation) to prevent or de-emphasize (where appropriate) the use of pharmaceutical interventions (Yassaie and Brooks, 2025). Such efforts can be quite impactful, given the emissions associated with pharmacotherapy (Kaur et al., 2025; Richie, 2022). Clinicians can also integrate conservation volunteering and sustainability awareness into clinical programming, which has been associated with improved mental health outcomes (Coventry et al., 2021).

Climate change is already a focus of concern in clinical services, and this will increase as the crisis worsens (Anderson et al., 2024; Pihkala, 2024). This challenges therapists to develop competence in addressing issues such as eco distress, eco anxiety, eco grief and “climate sorrow” (Anderson et al., 2024; Pihkala, 2022, 2024) and to engage with specific ethical considerations including self-reflection on internalized climate denial or disavowal (O'Gorman, 2024). Specific clinical populations might include environmental scientists impacted by vicarious trauma of climate breakdown (Pihkala, 2020) or activists (Lückemeier, 2025), including those in need of climate-aware therapists who do not inappropriately pathologize their concerns (O'Gorman, 2024). Competence might also include awareness of fostering adaptive coping for climate-aware individuals, including action, distancing and grieving (Pihkala, 2022) as well as delivering training for “mental health first response” in nature disasters (Anderson et al., 2024; James et al., 2016).

Psychologists should develop an understanding of community-based models of interventions that can be taken to scale to build resilience in the general population (cf. Monsell et al., 2021; Wray, 2022) (see Table 1 and Figure 1 for further examples). For example, the group-based “Living with the Climate Crisis” program assists participants to process emotions associated with climate change (Buchs et al., 2015). Further relevant resources are available through the websites of organizations such as the Climate Minds Coalition.

#### Health psychology

Health psychology emphasizes models of individual and systems change (de Castro and Reis, 2025; Papies et al., 2025), which can highlight important factors to target when attempting behavior change. Other highly useful skills that overlap with other subdisciplines include expertise in evidence synthesis, implementation science and intervention development (e.g., influencing individual behaviors within systems) (Papies et al., 2025). These skills are highly pertinent to supporting any collective efforts, for instance, for interpreting and applying the latest evidence in science communication, persuasion, and utilizing models for mobilizing and organizing.

#### Educational psychology

Children and youth are at increased risk for the impacts of climate change and have the least political power to address it. Perceived inaction from political leaders may lead to a sense of betrayal and moral injury in youth (Hickman, 2024). This is one reason they experience eco-anxiety (Galway and Field, 2023) and may conceptualize climate change differently from adults (Hickman, 2024; O'Hare, 2022). Interventions with this population should be modified accordingly (O'Hare, 2022). Valero (2025) overviews literature relevant to addressing climate distress in youth, including examples of projects that have been used to help with climate anxiety as well as actions by youth and schools (UNESCO, 2021). Educational psychologists can contribute to identifying, developing, implementing and evaluating training for educators to support students in emotional responses to concerns raised and to direct these into impactful action (UNESCO, 2021)—which should be free from influence by the fossil fuel industry (Keary and Chestnut, 2025).

#### Industrial/organizational psychology

Industrial and organizational (I–O) psychology expertise in change management, leadership engagement, work-life satisfaction, training, organizational culture and quality improvement can be also applied to improving sustainability within and outside of employment (Kühner et al., 2025; Maskell, 2026; Meyers and Rutjens, 2022).

There are emerging efforts to make health- and other organizations more sustainable (e.g., Duong, 2023). For instance, changes might include improving sustainability in food services through reducing food waste, increasing the presence of plant-rich menus (Morgenstern et al., 2024), and reducing the use of single-use items (McQuerry et al., 2021). Another focus has been in improving sustainability in procurement practices (Lavoie et al., 2024).

Within environmental organizations, I–O psychologists or psychologists applying evidence-informed I–O principles can inform strategies to address conflict management, recruitment and retention of activists, training in organizing/advocacy and evaluating both processes and outcomes (e.g., number of petition signatures; number of new sign-ups; environmental policy gains) and evidence-informed organizing models (c.f. Ganz, 2010). Resources for such organizing models are also readily available (Organizing: People, Power, Change, n.d.).

#### Community psychology

Community psychology's focus on context, collective action and systems change is highly applicable to climate mitigation and adaptation (Barnes et al., 2025). Community psychologists can contribute to efforts to build resiliency, promote community empowerment and promote social justice (Fernandes-Jesus et al., 2020; Lewis and Zlotowitz, 2021). Examples might include contributing psychological expertise to foster community resilience following climate events (Lewis and Zlotowitz, 2021), empowering communities to participate in local environmental initiatives or supporting efforts to generate economic models other than capitalism (Malherbe and Mavundla, 2025)—a prime driver of climate change.

#### Conservation psychology

Conservation psychology utilizes psychological science across subdisciplines to promote conservation behaviors (e.g., Clayton and Myers, 2015; Wallen and Landen, 2020). This includes understanding how people value nature, why they behave in environmentally harmful or beneficial ways, how to encourage pro-environmental behaviors, how to shape organizational practices, and how to engage stakeholders and communities. Crucially, more recently, conservation psychology has included efforts to collaborate across organizations including sharing resources and coordinating efforts (Maynard et al., 2022) and consider how to utilize organizing strategies to build political power (Jones and Niemiec, 2023). These strategies can be applied to shaping effective climate action.

#### Research, training, and academia

Research institutions, including universities, can contribute to addressing the climate crisis through research, training and collaborating with and providing support to community environmental groups. Involvement of universities is particularly relevant considering that research funding from the fossil fuel industry and other university activities may be associated with indirect obstruction on climate action (Gardner et al., 2022; Hiltner et al., 2024; Maina et al., 2020).

Consistent with consideration of the existential threat posed by the climate crisis, decision makers can heed the call of the editor of the British Medical Journal to prioritize funding for climate research (Auckland et al., 2022; Haines et al., 2020). Enacting this call would then be reflected in the policies of journals and funding bodies.

Previous publications have outlined a breadth of relevant research agendas for psychological research in relation to the climate crisis (e.g., American Psychological Association, 2022; Canadian Psychological Association, 2021; Nielsen et al., 2021; Nori-Sarma and Galea, 2024; Papies et al., 2025; Pearson et al., 2016). Papies et al. (2025) recommended prioritizing research that will lead to rapid emissions reduction, working in interdisciplinary research teams, utilizing behavior rather than intentions as dependent variables and including description of context that influences impact.

Vestergren et al. (2024) emphasized the critical importance of generating large, shared data sets and considering context when reporting outcomes of activism and collective action strategy (rather than being individually-focused). Lastly, recent updates include prioritizing research questions identified by climate activists, for instance in motivating others (Hamann et al., 2025). An important prospective role for research psychologists is intentionally finding ways to partner with climate organizations to improve their functioning and impact.

A final and emerging issue relevant to psychologists in research or academic positions is the use of artificial Intelligence-based (AI) technologies. AI has been described as “one of the world's most resource-intensive digital technologies” and consideration of the environmental impact of AI in research is therefore warranted (Fiske et al., 2025).

Whilst there are multiple avenues for potential research, it should be noted that while these will refine efforts, the urgency of the current situation means that it is important *not* to forestall action in the pursuit of more knowledge (cf. Dupont et al., 2025)—there is “No Research on a Dead Planet” (Thierry et al., 2023).

Universities are also major venues for training. Similar to other health professionals (e.g., medical training; cf. Cerceo et al., 2024; McKinnon et al., 2022), academics and students can advocate for curricula in psychology training to include consideration of planetary health, and do so across multiple courses (e.g., Aron, 2019). This includes across all courses/subdisciplines—including ethics, social-, cognitive-, organizational-, clinical-, and community psychology. Pedagogy could include competency, responsibilities, environmental racism within and outside the movement, science communication, addressing misinformation and disinformation, adaptation, mitigation, organizing and collective action (Nelson et al., 2025; also, see Table 1 for further examples) as well as ethical considerations such as the ethics of (in)action in the context of professional behavior (see Supplementary material A for vignettes^1^). Climate justice and action are also relevant to specific sub-disciplines within psychology, and thus its curricula, including clinical-, health-, educational-, industrial/organizational-, community-, and conservation psychology as well as ethics and jurisprudence (Papies et al., 2025).

### Individual action and advocacy

Based on Dupont et al.'s (2025) conceptualization of the response of scientists to the climate crisis, psychologists' actions can be organized along two dimensions: individual vs. collective advocacy/action; and work- vs. community-based advocacy/action. Individual action is broadly considered as an action that is undertaken by the individual with no structured effort to enlist others. Conversely, collective action/advocacy is an organizing strategy that focuses on systematic ways of building influence/power through enlisting others to work toward a common goal (McAlevey, 2016).

Whether or not the individual specifically identifies as a psychologist while engaging in climate-relevant activities, psychologists have specialized skills/knowledge/resources they may apply even when acting as private citizens (see examples in Figure 1). Further, professional ethics codes may apply if psychologists' actions as private citizens impact the reputation of psychology as a discipline, or if they raise questions regarding the individual's abilities to carry out responsibilities as a psychologist (e.g., Canadian Psychological Association, 2017).

Although individual behavior change alone is insufficient to shift carbon emissions at the scale necessary to mitigate catastrophic climate change, it has been identified as an important pathway to mitigation [Intergovernmental Panel on Climate Change (IPCC), 2023]. This includes contributing to a shift in social norms (Capstick et al., 2020; Fisher, 2024; Intergovernmental Panel on Climate Change (IPCC), 2023), creating conditions that support broader systemic change. Within this context, psychologists are well-positioned to contribute both as individual citizens and through their professional roles in shaping public understanding and behavior.

### Collective action and advocacy

The magnitude of action that is required to rapidly decarbonize and protect the environment and biodiversity loss is daunting. This is likely to worsen with the increasing use of AI-based technologies (c.f. Fiske et al., 2025), around which ethical considerations due to vast environmental and societal impact (water, energy, resource use, electronic waste, political destabilization) are only recently starting to be considered. Despite thousands of journal articles and studies conclusively illustrating climate change and environmental degradation, as well as numerous “calls to action” by psychologists, health professionals and academics, efforts to curb emissions and prevent or reverse environmental degradation and biodiversity loss have not been as impactful as necessary. Change in tactics and/or in the breadth of tactics should be considered (Racimo et al., 2022). There is a need to access, amplify and apply information relevant to building social movements. This includes collaborating across disciplines to understand political contexts and influences beyond contingencies around individual behaviors. When considering reasons for lack of progress, Gardner et al. (2022) comment:

Scientists must face a difficult truth that doesn't come easily to those of us who are most comfortable working diligently on experiments and journal articles: evidence alone, even if expertly communicated, is very easily ignored by those that do not wish to hear (para. 10).

**Figure 2:**
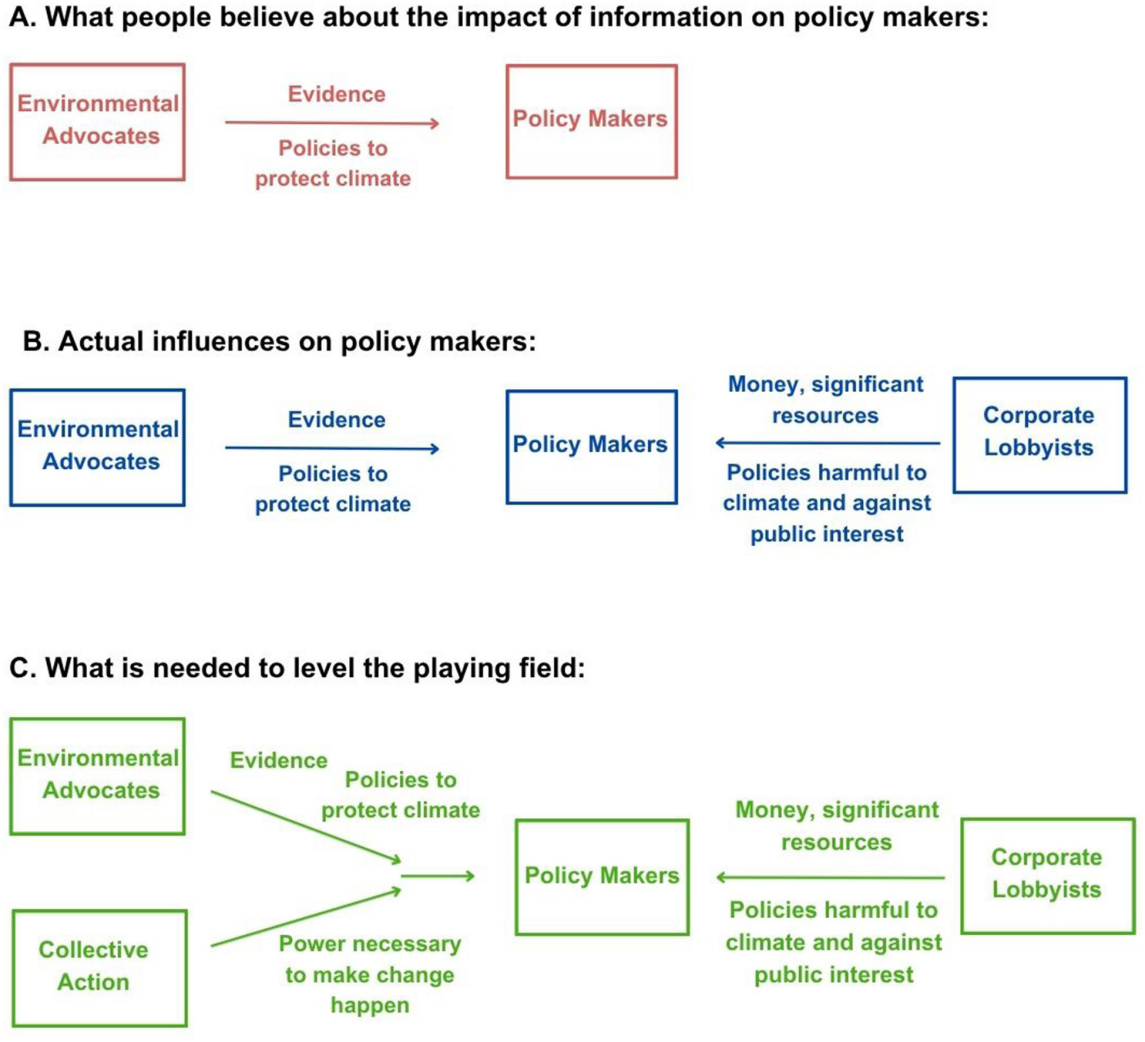
Conceptual diagram of influences on policy makers. Conceptual diagram illustrating the importance of collective action when attempting to influence policy makers. Adapted from (Gardner 2024), with the author's permission.

Whilst individual advocacy is commonly promoted as a way to influence decision makers, there are limitations to this tactic. Importantly, this landscape includes the influence of corporate lobbyists and those invested in protecting the status quo including through actively promoting disinformation to sway public opinion (e.g., Böhler et al., 2022; Brulle et al., 2021; Brulle, 2022; Goldberg et al., 2020; Hiltner et al., 2024; Klein, 2020; Lamb et al., 2020; Oreskes and Conway, 2011; Stoddard et al., 2021; and Figure 2).

Thus, while individual-level efforts and behavior change are helpful, the core of work should rather focus on engaging larger groups of individuals to mobilize collectively to effect systems change and to sustain that change enough to accrue systems level influence (Dupont et al., 2025; McAlevey, 2016; Vestergren et al., 2018, 2024). While many psychologists already engage in individual-level lifestyle changes, fewer engage in climate activism utilizing collective action (cf. Dablander et al., 2024), although such behavior can support individual behavior change (Vestergren et al., 2018) This is significant, since a recent review found that climate activism can influence policymakers and also voting behaviors (Thomas-Walters et al., 2025) and larger successful campaigns often start at the grassroots level (Nelson et al., 2025). There are growing calls from the scientist and health care professional communities to move from the sidelines to more strident and visible climate advocacy and activism (e.g., Dupont et al., 2025; Gardner et al., 2021; Maibach et al., 2021; Power et al., 2022; Urai and Kelly, 2023; Wyatt et al., 2024). Klein (2020) quotes Margaret Klein Salamon (director of The Climate Mobilization):

We are too late in the game for… individualism, the idea that “I'll take care of my emissions, you take care of yours.” What we envision is a rapid transition of our entire economy and society, with all hands on deck. (Klein, 2020, p. 3)

The United Nations' Intergovernmental Panel on Climate Change (IPCC) recognizes with “high confidence” that “collective action as part of social or lifestyle movements underpins system change” [Intergovernmental Panel on Climate Change (IPCC), 2023, p. 504]. Building strength in numbers, through collective action, is the primary advantage held by the majority to combat highly-resourced corporate interests (Gardner et al., 2021; Huber, 2022; McAlevey, 2016) (see Figure 2 for a conceptualization).

As mentioned, certain national ethical codes for psychologists directly or indirectly support taking actions to avert the climate crisis. What is needed in support of this is a model for action. Historically, social movements that have been effective have built sufficient “power” (i.e., political influence) through collective action to effect rapid and significant change, even when faced with entrenched vested interests (e.g., Engler and Engler, 2016; Ganz, 2010; McAlevey, 2016).

Social movements focused on sustained collective action allow for pooling resources (including knowledge, skills and experience in organizing) and can be encouraging and energizing for participants (cf. Gulliver et al., 2023). A host of tools, resources, free training and research synthesis developed from a long history of activism (predominantly in labor and civil rights, although also environmental movements), are available to guide and structure efforts to build collective action (see Supplementary material B). These resources provide accessible guidance on strategies and tactics for building movements. Psychologists can contribute to collective action by drawing on these resources and integrating them with their highly relevant “soft skills,” including alliance building, motivation, and conflict resolution and relational conversations and support, particularly useful in “relational organizing” (Jones and Niemiec, 2023).

Another important and sometimes overlooked skill psychologists bring to collective action is in data collection, program evaluation and ongoing quality improvement efforts within environmental organizations that have vast mandates and limited resources. Psychologists can partner with them to understand how to maximize effectiveness and can use data-based feedback loops to generate motivation and hope.

### Collective action in organizations/ at the workplace

Universities, educational, correctional, health, and professional practice bodies are organizations in their own right, with policies and practices that impact the degree to which they contribute to or alleviate greenhouse gas emissions (cf. Gardner et al., 2021). This is significant because institutions for which some psychologists work (e.g., health organizations) are major contributors to greenhouse gas emissions (Pichler et al., 2019; Tennison et al., 2021). These institutions also constitute large workforces that can collectively shift practice regarding financial investments and ultimately, power (Howard et al., 2023). Administrators, staff, academics and members can initiate (or resist) actions in alignment with social justice, including protective environmental behaviors and can do so individually or more impactfully, collectively.

Collective action can be facilitated through union- or professionally-based activism (e.g., Climate Minds Coalition, 2024; Das, 2023), as well as in coalitions across movements (Jasper, 2000) and within institutions (e.g., Nelson et al., 2025). The size and influence of health sector workers can be leveraged to shift culture in large segments of society (Howard et al., 2023). In the light of the crisis, employers should formally recognize and allow for contributions to social movements (Gardner et al., 2021) and this might be supported by unions.

Research has indicated that one barrier to professional participation in the climate crisis is concern for reputation or fear of employment ramifications (Gardner et al., 2021). Solidarity among union members can be powerful in protection from such concerns and in normalizing involvement in collective action (e.g., Tannock, 2024; Vogt and Subasinghe, 2025). This can include protection when psychologists or others participate in protest. In their position statement regarding the role of clinical psychologists in the climate crisis, the Association of Clinical Psychologists UK (ACP-UK) articulates their commitment to engage in “dialogue with … unions to call for timely and sensitive responses to those convicted for non-violent actions of moral protest …” (Morgan et al., 2020).

Examples of unions influencing legislation related to broader human rights, include efforts to decarbonize the economy (Myers, 2024; Thomas, 2021). There are examples of a professional health organization (Ontario Medical Association) being instrumental in winning protections in energy policy that reduces air pollution (Cundiff, 2015) and in efforts to reduce plastic use in health care settings (Waddington, 2025). Finally, there are examples of psychologist organizations specifically engaging in political action to influence climate policy. Acting in solidarity with other health professions, the ACP-UK generated an open letter endorsed by over 1,000 psychologists pointing out the psychological impacts of environmental emergencies, supporting peaceful protest as a reasonable choice and a recommendation to follow UN guidelines for a timeframe for action (Morgan et al., 2020). It is notable that this latter effort was working in solidarity with other efforts, that there was attention to accepted expertise regarding targeted action (using UN guidelines) and advocating for social justice (which is aligned with psychologist ethics and values). The letter articulated support for the necessity for peaceful protest in the context of actions in the UK by Extinction Rebellion and other groups that use non-violent civil disobedience as a tactic.

A practical way to make it easier for psychologists to undertake collective action for changes (cf. Luo et al., 2023) is to join existing efforts, including accessing and utilizing tools and resources and joining organizations that are relevant to psychologists' geographic and professional contexts. Supplementary material B provides a non-exhaustive list of organizations that may assist psychologists in engaging with climate action.

There are other examples of health workers (or students) acting collectively within their professional roles and networks, through tactics related to finances. Divesting pensions may be around 20 times as effective at reducing pension holders' carbon footprint as individual actions such as reducing air travel (Make My Money Matter, 2024). Health workers have collectively participated in economic and political pressure strategies (such as divestments or boycotts) targeting systems/industries inconsistent with their professional values (Maina et al., 2020). For instance, after recognizing the financial risks of not protecting the climate, there have been successful efforts to divest pensions from the fossil fuel industry by medical associations (National Academy of Medicine, 2023), across pension plans (e.g., SHIFT Action for Pension Wealth and Planet Health; Taylor, 2023) and universities' pension plans and endowment investments (Nelson et al., 2025). Other institutions (particularly universities) have acted to remove specific financial institutions from campus due to their involvement with funding fossil fuel projects (Amnesty International, 2022; Law, 2023).

### Urgent action and non-violent civil disobedience

No matter how well-informed or articulate the climate advocate, lobbyists and corporations can yield enormous influence over policy makers (Figure 2) (e.g., Environmental Defence, 2023; Goldberg et al., 2020; Hiltner et al., 2024; Klein, 2020). This has led to a reconsideration of tactics within climate activism (Racimo et al., 2022). O'Gorman (2024) notes that “to deal with the changing landscape psychologists need to revisit what it means to practice ethically” (p. 46). As a last resort, this has led some to broaden the use of available tactics to include non-violent civil disobedience (NVCD), also known as “disruptive action”. NVCD is defined by Tindall et al. (2008) as “a nonviolent action engaged in by an individual who refuses to obey a law for moral or philosophical reasons” (p. 22). Ethical dimensions of use of this tactic, including its efficacy, should be considered (see Supplementary material A for vignettes to discuss).

Large-scale disruptive action can be effective at increasing awareness and engagement in social movements (Kenward and Brick, 2024). Further, effective activism uses a range of tactics (Gulliver et al., 2021). As mentioned, NVCD is generally a last resort and incorporated alongside a diversity of other “low-risk” activities, such as advocacy or marches. Further, while for rapid changes disruption may be necessary, it is not always effective (Young and Thomas-Walters, 2024).

Use of NVCD as a tactic within an overall strategy to build power and pressure decision makers has also been used by some scientists and climate/health groups (e.g., Scientist Rebellion; Health for XR), alarmed and dismayed at government inaction and corporate interference in climate action. Scientists and health professionals have participated in public protests wearing white lab coats or scrubs as a way of emphasizing their identity and concern as professionals (Global March for Science, 2017; Health for XR, 2023) or in public-facing actions such as signing open letters appealing to decision-makers in their professional capacity (Morgan et al., 2020). Higher-risk (i.e., greater risk of being in conflict with the law) disruptive actions have included scientists gluing giant size research studies onto government agencies in protest at inaction (Gardner et al., 2022; Gayle, 2022). Academics and health professionals have incurred significant personal and professional costs for participating in these acts of conscience and have done so out of concern for the repercussions of climate breakdown (e.g., Buckley, 2023; Extinction Rebellion, 2022; Paxton and Jones, 2020; Yassaie, 2024).

NVCD has been endorsed by some academic and health professionals as an act of conscience related to climate or other issues (e.g., Auckland et al., 2022; Bennett et al., 2020; Capstick et al., 2020, 2022; Fossen, 2025; Gardner et al., 2021; Racimo et al., 2022; Rossa-Roccor et al., 2021; The Lancet, 2020). Engagement in NVCD, however, has received minimal attention within psychology to date. The ACP-UK has publicly articulated support for non-violent environmental activists, including clinical psychologists (Morgan et al., 2020). Clinical psychologists have participated in NVCD in the context of the climate crisis and their actions have been scrutinized within their professional associations (Cox, 2021; Paxton and Jones, 2020).

The Canadian code of professional ethics (IV.18) provides some guidance for ethical decision-making for psychologists to act outside of the law, including the importance of seeking “consensus as to the most ethical course of action and the most responsible, knowledgeable, effective, and respectful way to carry it out” (Canadian Psychological Association, 2017, p. 33) and to accept consequences of choices (Canadian Psychological Association, 2017, p. 33; Simmons, 2008). This echoes criteria for use of NVCD by regulated health professionals articulated by Bennett et al. (2020) including the consideration of the efficacy of (not) using the tactic as well as costs (including to the reputation of the profession) of (not) doing so (see Supplementary material A for vignettes exploring ethical dimensions).

O'Gorman (2024) identifies potential loss of employment and insurance as consequences in the UK where certain non-violent protest tactics are criminalized. The current authors had differing views on whether NVCD is a legitimate tactic to consider in the context of the climate crisis, with some supporting the thoughtful consideration of NVCD in some circumstances and others (FS and SC) believing that possible harm to the reputation of the profession is too high to consider the use of NVCD tactics.

## Conclusion

Given the scale, urgency, and wide-ranging impacts of the climate crisis on mental health and human wellbeing, there is a strong case for considering climate stewardship not as an optional or peripheral activity, but as a central component of contemporary psychological practice and professional responsibility. Psychologists not just can but should leverage their expertise and networks to influence decision-making, recognizing that the public policy arena includes powerful corporate interests working against needed change. By embracing their ethical responsibilities and actively participating in addressing the climate crisis—for which we provided numerous directions and specific examples—psychologists should significantly contribute to safeguarding planetary and societal wellbeing.

## Footnotes

1 These case vignettes are intended to illustrate how individual and collective climate stewardship may materialize in real-world contexts, highlighting both the enabling and constraining factors that can influence outcome, as well as the ethical considerations and dilemmas that may arise in practice. They are designed to support individual reflection or group discussion across settings, including undergraduate and postgraduate training, as well as in the continuing professional development of experienced practitioners.

## References

[B1] Abassi K. Ali P. Barbour V. Benfield T. Bibbins-Domingo K. Hancock S. . (2023). Time to treat the climate and nature crisis as one indivisible global health emergency. Lancet 402, 1603–1606. doi: 10.1016/S0140-6736(23)02289-4

[B2] Adams M. (2021). Critical psychologies and climate change. Curr. Opin. Psychol. 42, 13–18. doi: 10.1016/j.copsyc.2021.01.007 33636522

[B3] American Psychological Association (2017). Ethical Principles of Psychologists and Code of Conduct (2002, amended effective June 1, 2010, and January 1, 2017). Available online at: https://www.apa.org/ethics/code/ (Accessed May 8, 2026).

[B4] American Psychological Association (2022). Addressing the Climate Crisis: An Action Plan for Psychologists. Available online at: https://www.apa.org/science/about/publications/climate-crisis-action-plan.pdf (Accessed May 8, 2026).

[B5] Amnesty International (2022). RBC's Financing of the Coastal Gaslink Pipeline. Available online at: https://amnesty.ca/features/rbcs-financing-of-the-coastal-gaslink-pipeline/ (Accessed May 8, 2026).

[B6] Anderson J. Staunton T. O'Gorman J. Hickman C. (eds.) (2024). Being a Therapist in a Time of Climate Breakdown. London: Routledge. doi: 10.4324/9781003436096

[B7] APA Task Force on Climate Change (2022). Addressing the climate crisis: an action plan for psychologists (summary). Am. Psychol. 77, 799–811. doi: 10.1037/amp0001041 36037502

[B8] Arnout B. A. (2023). An epidemiological study of mental health problems related to climate change: a procedural framework for mental health system workers. Work 75, 813–835. doi: 10.3233/WOR-220040 36710698

[B9] Aron A. (2019). The climate crisis needs attention from cognitive scientists. Trends Cogn. Sci. 23, 903–906. doi: 10.1016/j.tics.2019.08.001 31492580

[B10] Association of Child Psychotherapists (2021). ACP Statement on the Climate Environmental Emergency. London: Association of Child Psychotherapists.

[B11] Atwoli L. Baqui A. Benfield T. Bosurgi R. Godlee F. Hancocks S. . (2021). Call for emergency action to limit global temperature increases, restore biodiversity, and protect health. Lancet 398, 939–941. doi: 10.1016/S0140-6736(21)01915-2 34496267 PMC8428481

[B12] Auckland C. Blumenthal-Barby J. Boyd K. Earp B. Frith L. Fritz McMillan, J. . (2022). Medical ethics and the climate change emergency. J. Med. Ethics 48, 939–940. doi: 10.1136/jme-2022-108738 36442972

[B13] Australian Psychological Society (2007). Australian Psychological Society Code of Ethics. Available online at: https://psychology.org.au/getmedia/d873e0db-7490-46de-bb57-c31bb1553025/aps-code-of-ethics.pdf (Accessed May 8, 2026).

[B14] Australian Psychological Society (2020). Psychology and Climate Change: Position Statement. Australian Psychological Society. Available online at: https://psychology.org.au/getmedia/c876613b-7f96-4456-8975-1a82190ec1d2/20aps-position_statement-psychology_climate-change.pdf (Accessed May 8, 2026).

[B15] Baird R. (2008). The Impact of Climate Change on Minorities and Indigenous Peoples. Minority Rights Group International. Available online at: https://minorityrights.org/app/uploads/2024/01/download-524-the-impact-of-climate-change-on-minorities-and-indigenous-peoples.pdf (Accessed May 8, 2026).

[B16] Ballew M. T. Thomas-Walters L. Goldberg M. H. Verner M. Lu J, Marshall, J. Rosenthal S. A. . (2025). Climate change messages can promote support for climate action globally. Glob. Environ. Change 90:102951. doi: 10.1016/j.gloenvcha.2024.102951

[B17] Barnes B. R. Fernandes-Jesus M. Trott C. D. Barnwell G. (2025) *Community, Psychology Climate Justice*. Cham: Springer Nature. doi: 10.1007/978-3-031-99223-0

[B18] Beauchamp T. L. Childress J. F. (2019). Principles of Biomedical Ethics, 8th Edn. New York, NY: Oxford University Press.

[B19] Bennett H. Macmillan A. Jones R. Blaiklock A McMillan J. (2020). Should health professionals participate in civil disobedience in response to the climate change health emergency? Lancet 395, 304–308. doi: 10.1016/S0140-6736(19)32985-X 31818491

[B20] Bhopal A. (2023). “Are you a researcher or an activist?”: navigating tensions in climate change and health research. J. Clim. Change Health 14:100267. doi: 10.1016/j.joclim.2023.100267

[B21] Bingley W. J. Tran A. Boyd C. P. Gibson K. Kalokerinos E. K. Koval, P. Kashima Y. . (2022). A multiple needs framework for climate change anxiety interventions. Am. Psychol. 77, 812–821. doi: 10.1037/amp0001012 35587891

[B22] Böhler H. Hanegraaff M. Schulze K. (2022). Does climate advocacy matter? The importance of competing interest groups for national climate policies. Clim. Policy, 22, 961–975. doi: 10.1080/14693062.2022.2036089

[B23] Brennan M. E. Madden D. L. (2023). The evolving call to action for including climate change and environmental sustainability themes in health professional education: a scoping review. J. Clim. Change Health 9:100200. doi: 10.1016/j.joclim.2022.100200

[B24] British Psychological Society (2021). Code of Ethics and Conduct. Available online at: https://explore.bps.org.uk/content/report-guideline/bpsrep.2021.inf94 (Accessed May 8, 2026).

[B25] British Psychological Society (2023). The Climate and Ecological Crisis: BPS Position Statement. Available online at: https://cms.bps.org.uk/sites/default/files/2023-10/BPS%20Position%20Statement%20on%20the%20Climate%20and%20Ecological%20Crisis.pdf (Accessed May 8, 2026).

[B26] Brulle R. J. (2022). Advocating inaction: a historical analysis of the Global Climate Coalition. Env. Polit. 32, 185–206. doi: 10.1080/09644016.2022.2058815

[B27] Brulle R. J. Hall G. Loy L. Schell-Smith K. (2021). Obstructing action: foundation funding and US climate change counter-movement organizations. Clim. Change 166:17. doi: 10.1007/s10584-021-03117-w

[B28] Buchs M. Hinton E Smith G. (2015). ‘It helped me sort of face the end of the world': the role of emotions for third sector climate change engagement initiatives. Environ. Values 24, 621–640. doi: 10.3197/096327115X14384223590177

[B29] Buckley C. (2023). After refusing to fly, a climate researcher loses his job. The New York Times. Available online at: https://www.nytimes.com/2023/10/12/climate/climate-researcher-no-fly.html (Accessed May 8, 2026).

[B30] Canadian Association of University Teachers (2017). Climate Change and Collective Bargaining. CAUT Bargain Advisory. Available online at: https://council.caut.ca/sites/default/files/10._ij_doc_4_caut-bargaining-advisory-climate-change-and-collective-bargaining-2017-05_0.pdf (Accessed May 8, 2026).

[B31] Canadian Medical Association (n.d.). Why Canada Needs a Net-Zero Health System. Canadian Medical Association. Available online at: https://www.cma.ca/our-focus/net-zero-emissions-health-system/why-canada-needs-net-zero-health-system#:~:text=Right%20now%2C%20however%2C%20the%20Canadian,across%20the%20continuum%20of%20care (Accessed May 8, 2026).

[B32] Canadian Psychological Association (2017). Canadian Code of Ethics for Psychologists, 4th Edn. Available online at: https://cpa.ca/docs/File/Ethics/CPA_Code_2017_4thEd.pdf (Accessed May 8, 2026).

[B33] Canadian Psychological Association (2021). Addressing Climate Change in Canada: The Importance of Psychological Science. Available online at: https://cpa.ca/docs/File/Position/ClimateChange_TASKFORCE_POSITIONPAPER_FINAL%20FEB42021.pdf (Accessed May 8, 2026).

[B34] Capstick S. Khosla R. Wang S. (2020). Bridging the Gap—The Role of Equitable Low-Carbon Lifestyles. In The Emissions Gap Report 2020. United Nations Environmental Programme. Available online at: https://orca.cardiff.ac.uk/id/eprint/138487/1/EGR20ch6.pdf (Accessed May 8, 2026).

[B35] Capstick S. Thierry A. Cox E. Berglund O. Westlake S. Steinberger J. (2022). Civil disobedience by scientists helps press for urgent climate action. Nat. Clim. Change 12, 773–774. doi: 10.1038/s41558-022-01461-y

[B36] Cerceo E. Cohen K. Hunter K. Hofstedt M. Kalwaney S. (2024). Serving up climate education: an innovative resident curriculum addressing climate change through plant-based solutions. J. Clim. Change Health 20:100330. doi: 10.1016/j.joclim.2024.100330 PMC12851175 41647121

[B37] Clayton S. (2024). A social psychology of climate change: progress and promise. Br. J. Soc. Psychol. 63, 1535–1546. doi: 10.1111/bjso.12749 38676432

[B38] Clayton S. Myers G. (2015). Conservation Psychology: Understanding and Promoting Human Care for Nature. Chichester: John Wiley and Sons.

[B39] Clayton S. Parnes M. F. (2025). Anxiety and activism in response to climate change. Curr. Opin. Psychol. 62:101996. doi: 10.1016/j.copsyc.2025.101996 39889454

[B40] Cleland S. E, Wyatt, L. H. Wei L. Paul N. Serre M. L. West J. J. Henderson S. B. . (2022). Short-term exposure to wildfire smoke and PM2.5 and cognitive performance in a brain-training game: a longitudinal study of U.S. Adults Environ. Health Perspect. 130:67005. doi: 10.1289/EHP10498 35700064 PMC9196888

[B41] Climate Minds Coalition (2024). Open Letter to All Parliamentarians. Climate Minds Coalition. Available online at: https://www.climatemindscoalition.com/openletter (Accessed May 8, 2026).

[B42] Conner J. Crawford E. Galioto M. (2023). The mental health effects of student activism: persisting despite psychological costs. J. Adolesc. Res. 38, 80–109. doi: 10.1177/07435584211006789

[B43] Coventry P. A. Brown J. E. Pervin J. Brabyn S. Pateman R. Breedvelt J. . (2021). Nature-based outdoor activities for mental and physical health: systematic review and meta-analysis. SSM Popul. Health 16:100934. doi: 10.1016/j.ssmph.2021.100934 34646931 PMC8498096

[B44] Cox P. (2021). The HCPC should support climate change activists, not question their fitness to practice. Clin. Psychol. Forum 346, 36–42. doi: 10.53841/bpscpf.2021.1.346.36

[B45] Crane K. Li L. Subramanian P. Rovit E. Liu J. (2022). Climate change and mental health: a review of empirical evidence, mechanisms and implications. Atmosphere 13:2096. doi: 10.3390/atmos13122096 37727770 PMC10508914

[B46] Cundiff B. (2015). Ontario's Coal Phase Out. Ontario's Clean Air Alliance. Available online at: https://www.cleanairalliance.org/wp-content/uploads/2015/04/CoalPhaseOut-web.pdf (Accessed May 8, 2026).

[B47] Dablander F. Sachisthal M. S. M. Cologna V. Strahm N. Bosshard A. Grüning N.-M. . (2024). Climate change engagement of scientists. Nat. Clim. Chang. 14, 1033–1039. doi: 10.1038/s41558-024-02091-2

[B48] Darwell S. (ed.). (2002). Deontology. Oxford: Wiley-Blackwell.

[B49] Das A. (2023). Does unionization reduce CO2 emissions in Canada? Environ. Sci. Pollut. Res. 30, 61455–61465. doi: 10.1007/s11356-022-19301-z PMC8891740 35239115

[B50] de Castro E. K. Reis M. (2025). Contributions of health psychology to climate change: a review. Int. J. Environ. Res. Public Health 22:634. doi: 10.3390/ijerph22040634 40283857 PMC12026601

[B51] Dixon B. Tassell-Matamua N. Ana O. B. S. Reyes M. E. S. (2022). “The Asia-Pacific climate crisis and psychology's responses,” in Climate Action and Global Psychology, eds. A. Clinton, B. Dixon, and T. Morrisey (Wellington: New Zealand Psychological Society), 69–86.

[B52] Driscoll D. (2019). When ignoring the news and going hiking can help you save the world: environmental activist strategies for persistence. Sociol. Forum 35, 189–206. doi: 10.1111/socf.12573

[B53] Driver J. (2012). Consequentialism. London: Routledge doi: 10.4324/9780203149256

[B54] Duong D. (2023). How Canadian hospitals are decreasing carbon emissions. Can. Med. Assoc. J. 195:E594. doi: 10.1503/cmaj.1096048 37094870 PMC10125190

[B55] Dupont L. Jacob S. Philippe H. (2025). Scientist engagement and the knowledge–action gap. Nat. Ecol. Evol. 9, 23–33. doi: 10.1038/s41559-024-02535-0 39304789

[B56] Engler M. Engler P. (2016). This Is an Uprising: How Nonviolent Revolt Is Shaping the 21st Century. New York, NY: Nation Books.

[B57] Environmental Defence (2023). Big Oil's Big Year: A Summary of Big Oil's 2023 Federal Lobbying [Report]. Available online at: https://environmentaldefence.ca/wp-content/uploads/2024/08/Report_Big-Oils-Big-Year-2023-2.pdf (Accessed May 8, 2026).

[B58] Extinction Rebellion (2022). 13 Scientists Held in Prison After Nonviolent Climate Protest in Germany. Available online at: https://www.pressenza.com/2022/10/munich13-scientists-held-in-prison-after-nonviolent-climate-protest-in-germany/ (Accessed May 8, 2026).

[B59] Faerron Guzmán C. A. Revers N. Ji J. S. Lacey-Hall O. Mahmood J. Masztalerz O. . (2025). Planetary health: focusing on the Intersection of Human Health and the Earth System. Annu. Rev. Environ. Resour. 50, 303–337. doi: 10.1146/annurev-environ-111523-102309

[B60] Fernandes-Jesus M. Barnes B. Farias Diniz R. (2020). Communities reclaiming power and social justice in the face of climate change. Commun. Psychol. Glob. Perspect. 6, 1–21. doi: 10.1285/i24212113v6i2-2p1

[B61] Finnerty S. Piazza J. Levine M. (2024). Scientists' identities shape engagement with environmental activism. Commun. Earth Environ. 5:240. doi: 10.1038/s43247-024-01412-9

[B62] Finnis J. (2011). Natural Law and Natural Rights. Oxford: Oxford University Press.

[B63] Fisher D. R. (2024). Saving Ourselves: From Climate Shocks to Climate Action. Columbia University Press. doi: 10.7312/fish20930

[B64] Fiske A. Radhuber I. Willem T. Buyx A. Celi L. McLennan S. (2025). Climate change and health: the next challenge of ethical AI. Lancet Glob. Health 13:e1314–e1320. doi: 10.1016/S2214-109X(25)00124-X 40580996

[B65] Fossen T. (2025). Academic activism and the climate crisis: should scholars protest? *Perspect. Polit*. 1–16. doi: 10.1017/S1537592725000350

[B66] Galway L. P. Field E. (2023). Climate emotions and anxiety among young people in Canada: a national survey and call to action. J. Clim. Change Health 9:100204. doi: 10.1016/j.joclim.2023.100204

[B67] Ganz M. (2010). “Leading change: leadership, organization, and social movements,” in Handbook of Leadership Theory and Practice, eds. N. Nohria, and R. Khurana (Boston, MA: Harvard Business Press), 1–10.

[B68] Gardner C. J. (2024). What Some People Think the Public Policy Arena Looks Like [Post]. Available online at: https://www.linkedin.com/search/results/all/?heroEntityKey=urn%3Ali%3Afsd_profile%3AACoAABetdOkB7OQQu2Xg6SZcMTVe_6_OAWL7vkAandkeywords=Charlie%20J.%20Gardnerandorigin=ENTITY_SEARCH_HOME_HISTORYandsid=e8! (Accessed September 27, 2026).

[B69] Gardner C. J. Cox E. Capstick S. (2022). Extinction Rebellion Scientists: Why We Glued Ourselves to a Government Department? The Conversation. Available online at: https://theconversation.com/extinction-rebellion-scientists-why-we-glued-ourselves-to-a-government-department-181799 (Accessed May 8, 2026).

[B70] Gardner C. J. Thierry A. Rowlandson W. Steinberger J. K. (2021). From publications to public actions: the role of universities in facilitating academic advocacy and activism in the climate and ecological emergency. Front. Sustain. 2:679019. doi: 10.3389/frsus.2021.679019

[B71] Gayle D. (2022). XR scientists glue hands to business department in London climate protest. The Guardian. Available online at: https://www.theguardian.com/environment/2022/apr/13/xr-scientists-glue-hands-to-business-department-in-london-climate-protest (Accessed May 8, 2026).

[B72] Gifford R. (2011). The dragons of inaction: psychological barriers that limit climate change mitigation and adaptation. Am. Psychol. 66, 290–302. doi: 10.1037/a0023566 21553954

[B73] Gifford R. (2021). New dragons of inaction discovered. Psynopsis 43, 26–27.

[B74] Gimalova M. Milton M. (2019). Taking action on climate change and environmental degradation. Psychologist 32, 2–3.

[B75] Global March for Science (2017). “Global March for Scienceraises concern over Trump policy.” *CBC News*, April 22. Available online at: https://www.cbc.ca/news/science/march-for-science-1.4081389 (Accessed May 8, 2026).

[B76] Godden N. Farrant B. Farrant J. Heyink E. Collins E. Tabeshfar M. . (2022). Climate change, activism, and supporting the mental health of children and young people: perspectives from Western Australia. J. Paediatr. Child Health 57, 1759–1764. doi: 10.1111/jpc.15649 PMC9299447 34792244

[B77] Goldberg M. H. Marlon J. R. Wang X. Leiserowitz A. (2020). Oil and gas companies invest in legislators that vote against the environment. PNAS 117, 5111–5112. doi: 10.1073/pnas.1922175117 32094171 PMC7071911

[B78] Gorski P. C. (2015). Relieving burnout and the ‘martyr syndrome' among social justice education activists: the implications and effects of mindfulness. Urban Rev. 47, 696–716. doi: 10.1007/s11256-015-0330-0

[B79] Green J. (2020). Less talk, more walk: why climate change demands activism in the academy. Daedalus 149, 151–162. doi: 10.1162/daed_a_01824

[B80] Greer F. (2023). Leveraging environmental assessment and environmental justice to deliver equitable, decarbonized built infrastructure. Environ. Res. Infrastruct. Sustain. 3:040401. doi: 10.1088/2634-4505/ad084b

[B81] Gulliver R. E. Pittaway C. Fielding K. S. Louis W. R. (2023). Resources that help sustain environmental volunteer activist leaders. Voluntas 34, 1299–1309. doi: 10.1007/s11266-023-00561-3 PMC9976682 37360504

[B82] Gulliver R. E. Star C. Fielding K. S. Louis W. R. (2022). A systematic review of the outcomes of sustained environmental collective action. Environ. Sci. Policy 133, 180–192. doi: 10.1016/j.envsci.2022.03.020

[B83] Gulliver R. E. Wibisono S. Fielding K. S. Louis W. R. (2021). The Psychology of Effective Activism. Cambridge: Cambridge University Press. doi: 10.1017/9781108975476

[B84] Haines A. Scheelbeek P. Abbasi K. (2020). The health case for urgent action on climate change. Br. Med. J. 368:m1103. doi: 10.1136/bmj.m1103 32229547 PMC7190277

[B85] Hamann K. R. S. Wenzel K. Dasch S. Junge E. von Agris A.-S. Bleh J. (2025). How can psychological research support movements for socio-ecological change? A qualitative study on psychological challenges and questions of activists. Glob. Environ. Psychol. 3:e13089. doi: 10.5964/gep.13089

[B86] Hantel A. Senay E. Richie C. Revette A. Nava-Coulter B. Hlubocky F. J. . (2024). A focus group study of ethical issues during climate-informed health decision-making. Nat. Clim. Chang. 14, 1040–1046. doi: 10.1038/s41558-024-02121-z

[B87] Harris F. (2023). Scientists deliver ‘final warning' on climate crisis: act now or it's too late. The Guardian. Available online at: https://www.theguardian.com/environment/2023/mar/20/ipcc-climate-crisis-report-delivers-final-warning-on-15c (Accessed May 8, 2026).

[B88] Hayes K. Berry P. Ebi K. L. (2019). Factors influencing the mental health consequences of climate change in Canada. Int. J. Environ. Res. Public Health 16:1583. doi: 10.3390/ijerph16091583 31064134 PMC6539500

[B89] Health for XR (2023). 21st-24th April 2023: Hundreds of Health Professionals Join 100,000 People at the Big One. Health for extinction rebellion. Available online at: https://healthforxr.com/21st-24th-april-hundreds-of-health-professionals-join-100000-people-at-the-big-one/ (Accessed May 8, 2026).

[B90] Henritze E. Goldman S. Simon S. Brown A. D. (2023). Moral injury as an inclusive mental health framework for addressing climate change distress and promoting justice-oriented care. Lancet Planet. Health 7, e238–e241. doi: 10.1016/S2542-5196(22)00335-7 36889865

[B91] Hickman C. (2024). Eco-anxiety in children and young people – a rational response, irreconcilable despair, or both? Psychoanal. Study Child 77, 356–368. doi: 10.1080/00797308.2023.2287381

[B92] Hiltner S. Eaton E. Healy N. Scerri A. Stephens J. C. Supran G. (2024). Fossil fuel industry influence in higher education: a review and a research agenda. WIREs Clim. Change 15:e904. doi: 10.1002/wcc.904

[B93] Howard C. MacNeill A. J. Hughes F. Alqodmani L. Charlesworth K. Almeida R. . (2023). Learning to treat the climate emergency together: social tipping interventions by the health community. Lancet 7, E251–E264. doi: 10.1016/S2542-5196(23)00022-0 36889866

[B94] Huber M. T. (2022). Climate Change as Class War: Building Socialism on a Warming Planet. London: Verso.

[B95] Hursthouse R. (2017). “On virtue ethics,” in Applied Ethics. A Multicultural Approach, ed. L. May (London: Routledge), 29–35. doi: 10.4324/9781315097176-5

[B96] Intergovernmental Panel on Climate Change (IPCC) (ed.) (2023). “Demand, services and social aspects of mitigation,” in Climate Change 2022—*Mitigation of Climate Change: Working Group III Contribution to the Sixth Assessment Report of the Intergovernmental Panel on Climate Change* (Cambridge: Cambridge University Press), 503–612. doi: 10.1017/9781009157926.007

[B97] Ivanova D. Barrett J. Wiedenhofer D. Macura B. Callaghan M. Creutzig F. (2020). Quantifying the potential for climate change mitigation of consumption options. Environ. Res. Lett. 15:093001. doi: 10.1088/1748-9326/ab8589

[B98] James L. E. Welton-Mitchell C. Moun S. L. (2016). Community-Based Disaster Mental Health Intervention (CBDMI): Curriculum Manual for Use with Communities Affected by Natural Disasters in Haiti. http://www.elrha.org/researchdatabase/community-based-disaster-mental-health-intervention-cbdmhi-curriculum-manual-use-communities-affected-natural-disasters-haiti/

[B99] Jasper J. M. (2000). Review of coalitions across the class divide: lessons from the labor, peace, and environmental movements, by F. Rose. Soc. Forces 79, 790–791. doi: 10.2307/2675520

[B100] Jones M. S. Niemiec R. M. (2023). Motivating relational organizing behavior for biodiversity conservation. Conserv. Sci. Pract. 5:e12880. doi: 10.1111/csp2.12880

[B101] Jos P. H. (2006). Social contract theory: implications for professional ethics. Am. Rev. Public Adm. 36, 139–155. doi: 10.1177/0275074005282860

[B102] Kałwak W. Ekelund B. Gale N. Peter F. Wortelboer S. (eds.) (2024). Climate Crisis and the Human Factor: 10 Psychological Keys to Unlocking Climate Action. Opinion Paper of the EFPA's Expert Reference Group for Psychology and Climate Change. Brussels: European Federation of Psychologists' Associations EFPA AISBL.

[B103] Kaur H. Parascandalo F. Krantzberg G. Ko E. Mathur N. Gill A. S. . (2025). The journey of a pill. Can. Fam. Physician 71:298. doi: 10.46747/cfp.7104263 40228876 PMC12007625

[B104] Keary A. Chestnut J. (2025). Polluting Education: The Influence of Fossil Fuels on Children's Education in Canada. Toronto, ON: Canadian Association of Physicians for the Environment and For Our Kids.

[B105] Kenner D. (2019). Carbon Inequality: The Role of the Richest in Climate Change. London: Routledge. doi: 10.4324/9781351171328

[B106] Kenward B. Brick C. (2024). Large-scale disruptive activism strengthened environmental attitudes in the United Kingdom. Glob. Environ. Psychol. 2:e11079. doi: 10.5964/gep.11079

[B107] Klein S. (2020). A Good War: Mobilizing Canada for the Climate Emergency. Toronto, ON: ECW Press.

[B108] Kleres J. Wettergren Å. (2017). Fear, hope, anger, and guilt in climate activism: social movement studies. Soc. Mov. Stud. 16, 507–519. doi: 10.1080/14742837.2017.1344546

[B109] Knight K. (2020). Climate activism as a clinical psychologist. Clin. Psychol. Forum 1, 40–44. doi: 10.53841/bpscpf.2020.1.332.40

[B110] Kühner C. Hüffmeier J. Zacher H. (2025). Environmental sustainability at work: it's time to unleash the full potential of industrial and organizational psychology. Ind. Organ. Psychol. 18, 466–506. doi: 10.1017/iop.2025.10015

[B111] Lamb W. F. Mattioli G. Levi S. Roberts J. T. Capstick S. Creutzig F. . (2020). Discourses of climate delay. Glob. Sustain. 3:e17. doi: 10.1017/sus.2020.13

[B112] Latter B. Demski C. Capstick S. (2024). Wanting to be part of change but feeling overworked and disempowered: researchers' perceptions of climate action in UK universities. PLoS Clim. 3:e0000322. doi: 10.1371/journal.pclm.0000322

[B113] Lavoie D. C. T. Maraj A. Wong G. Y. C. Parascandalo F. Sergeant M. (2024). Healthcare procurement in the race to net-zero: practical steps for healthcare leadership. Healthc. Manag. Forum 37, 384–389. doi: 10.1177/08404704241258152 PMC11348617 39033434

[B114] Law S. (2023). Lakehead University students demand school cut ties with RBC for funding fossil fuel projects. CBC News. Available online at: https://www.cbc.ca/news/canada/thunder-bay/lakeheaduniversity-rbc-fossilfuels-protest-1.6766076 (Accessed May 8, 2026).

[B115] Lee M. R. Harber M. (2025). The climate crisis is a global health emergency: a call to arms. Future Healthc. J. 12:100238. doi: 10.1016/j.fhj.2025.100238 40236927 PMC11998292

[B116] Lewis C. Zlotowitz S. (2021). How can our community psychology values and experience inform our response to the climate emergency. Clin. Psychol. Forum 346, 104–109. doi: 10.53841/bpscpf.2021.1.346.104

[B117] Li C. Lawrance E. L. Morgan G. Brown R. Greaves N. Krzanowski J. . (2022). The role of mental health professionals in the climate crisis: an urgent call to action. Int. Rev. Psychiatry 34, 563–570. doi: 10.1080/09540261.2022.2097005 36165755

[B118] Li N. Su Y. F. (2018) Message framing climate change communication: a meta analytical review. J. Appl. Commun. 102, 1–14. doi: 10.4148/1051-0834.2189

[B119] Lilienfeld E. Nicholas P. Breakey S. Corless I. (2018). Addressing climate change through a nursing lens within the framework of the United Nations Sustainable Development Goals. Nurs. Outlook 66, 482–494. doi: 10.1016/j.outlook.2018.06.010 30172574

[B120] Løkken Nordrum O. Mobacke I. MäNnistö T. Schønemann-Lund M, Björnsson, H. M. Sviland L. (2025). The climate crisis is a health crisis: a Statement by Nordic Doctors for Planetary Health and Climate Action. Scand. J. Public Health 53, 223–224. doi: 10.1177/14034948241302923 40079528 PMC11907728

[B121] Lord G. Reilly H. Löffler-Stastka H. (2025). Activist burnout among climate justice activists in Austria: an interpretative phenomenological analysis. Healthcare 13:2045. doi: 10.3390/healthcare13162045 40868661 PMC12385306

[B122] Lowe S. R. Bonumwezi J. L. Valdespino-Hayden Z. Galea S. (2018). Posttraumatic Stress and Depression in the Aftermath of Environmental Disasters: A Review of Quantitative Studies Published in 2018. Curr. Environ. Health Rep. 6, 344–360. doi: 10.1007/s40572-019-00245-5 31487033

[B123] Lückemeier C. L. (2025). Feeling the pain of the Earth: an intuitive inquiry into supporting climate activists with eco-grief. Conscious. Spiritual. Transpers. Psychol. 6, 17–34. doi: 10.53074/cstp.2025.89

[B124] Luo Y. Li A. Soman D. Zhao J. (2023). A meta-analytic cognitive framework of nudge and sludge. R. Soc. Open Sci. 10:230053. doi: 10.1098/rsos.230053 38034123 PMC10685127

[B125] MacPherson C. Wynia M. (2017). Should health professionals speak up to reduce the health risks of climate change? AMA J. Ethics 19, 1202–1210. doi: 10.1001/journalofethics.2017.19.12.msoc1-1712 29278346

[B126] Mahendran R. Xu R. Li S. Guo Y. (2021). Interpersonal violence associated with hot weather. Lancet Planet. Health 5, e571–e572. doi: 10.1016/S2542-5196(21)00210-2 34508676

[B127] Maibach E. Frumkin H. Ahdoot S. (2021). Health professionals and the climate crisis: trusted voices, essential roles. World Med. Health Policy 13, 137–145. doi: 10.1002/wmh3.421

[B128] Maina N. M. Murray J. McKenzie M. (2020). Climate change and the fossil fuel divestment movement in Canadian higher education: the mobilities of actions, actors, and tactics. J. Clean. Prod. 253:119874. doi: 10.1016/j.jclepro.2019.119874

[B129] Make My Money Matter (2024). UK Pensions: Climate Action Report an Analysis of Leading Pension Providers' Action on Climate Change. Available online at: https://makemymoneymatter.co.uk/wp-content/uploads/2024/02/Make-My-Money-Matter-Climate-Action-Report-2024.pdf (Accessed May 8, 2026).

[B130] Malherbe N. Mavundla B. (2025). “Resisting capitalism, resisting climate change: community psychology in and against the capitalocene,” in Community, Psychology and Climate Justice. Community Psychology, eds. B. R. Barnes, M. Fernandes-Jesus, C. D. Trott, and G., Barnwell (Cham: Springer), 99–114. doi: 10.1007/978-3-031-99223-0_6

[B131] Maskell J. (2026). Work Psychology and the Climate Crisis: A Handbook of Principles and Practice. London: Routledge. doi: 10.4324/9781003422631

[B132] Maynard L. Howorth P. Daniels J. Bunney K. L. Snyder R. Jenike D. . (2022). Conservation psychology strategies for collaborative planning and impact evaluation. Zoo Biol. 41, 425–438. doi: 10.1002/zoo.21692 35412653 PMC9790717

[B133] McAlevey J. (2016). No Shortcuts: Organising for Power in the New Gilded Age. Oxford: Oxford University Press. doi: 10.1093/acprof:oso/9780190624712.001.0001

[B134] McCowan T. (2020). The Impact of Universities on Climate Change: A Theoretical Framework. Transforming Universities for a Changing Climate. Working Paper Series No. 1, Institute of Education. Available online at: https://www.researchcghe.org/publication/the-impact-of-universities-on-climate-change-a-theoretical-framework/ (Accessed May 8, 2026).

[B135] McCunn L. J. Gillis P. Lavallee L. Houlding C. Perry A. Stronik M. . (2025). A Survey of Canadian Psychology Practitioners' Perceptions of Climate Change Advocacy—A Brief Report Composed for the Canadian Psychological Association by the Climate Change Advocacy Working Group [report]. Ottawa, ON: Canadian Psychological Association.

[B136] McKinnon S. Breakey S. Fanuele J. R. Kelly D. Eddy E. Tarbet A. . (2022). Roles of health professionals in addressing health consequences of climate change in interprofessional education: a scoping review. J. Clim. Change Health 5, 2667–2782. doi: 10.1016/j.joclim.2021.100086

[B137] McQuerry M. Easter E. Cao A. (2021). Disposable versus reusable medical gowns: a performance comparison. Am. J. Infect. Control 49, 563–570. doi: 10.1016/j.ajic.2020.10.013 33091509 PMC7572274

[B138] Mendy L. Karlsson M. Lindvall D. (2024). Counteracting climate denial: a systematic review. Public Underst. Sci. 33, 504–520. doi: 10.1177/09636625231223425 38243813 PMC11056086

[B139] Meyers M. C. Rutjens D. (2022). Applying a positive (organizational) psychology lens to the study of employee green behavior: a systematic review and research agenda. Front. Psychol. 13:840796. doi: 10.3389/fpsyg.2022.840796 35558698 PMC9087848

[B140] Monsell A. Krzanowski J. Page L. Cuthbert S. Harvey G. (2021). What mental health professionals and organisations should do to address climate change. Br. J. Psychol. Bull. 45, 215–221. doi: 10.1192/bjb.2021.17 PMC8499631 33947498

[B141] Morgan G. Snell T. Randall J. (2020). ACP-UK Statement on the Need for Action to Address the Climate Crisis. Association of Clinical Psychologists UK. Available online at: https://acpuk.org.uk/climate_change_statement/ (Accessed May 8, 2026).

[B142] Morgenstern S. Redwood M. Herby A. (2024). An innovative program for hospital nutrition. Am. J. Lifestyle Med. 19, 320–323. doi: 10.1177/15598276241283158 39540172 PMC11556658

[B143] Myers K. (2024). In 2023, unions became core to the climate movement. Canada's National Observer. Available online at: https://www.nationalobserver.com/2024/01/12/news/2023-unions-became-core-climate-movement (Accessed May 8, 2026).

[B144] Narayan S. Conway M. Rudolph L. Miller J. Linou N. Shreedhar J (2026). The imperative to counter fossil fuel industry disinformation for public health. Lancet Planet. Health 10:101435. doi: 10.1016/j.lanplh.2026.101435 41974144

[B145] National Academy of Medicine (2023). Statement on NAM Commitment to Reducing Greenhouse Gas Emissions. Available online at: https://nam.edu/statement-on-nam-commitment-to-reducing-greenhouse-gas-emissions/ (Accessed May 8, 2026).

[B146] Nelson M. Gere C. Cooper A. Thackray V. G. Aron A. R. (2025). The wins of the grassroots climate movement in the University of California. Front. Educ. 10:1491439. doi: 10.3389/feduc.2025.1491439

[B147] New Zealand Psychological Society (2002). Code of Ethics for Psychologists working in Aotearoa/New Zealand. Available online at: https://www.psychology.org.nz/members/professional-resources/code-ethics (Accessed May 8, 2026).

[B148] Nielsen K. S. Clayton S Stern P. C Dietz T Capstick S Whitmarsh L. (2021). How psychology can help limit climate change. Am. Psychol. 76, 130–144. doi: 10.1037/amp0000624 32202817

[B149] Nori-Sarma A. Galea S. (2024). Climate change and mental health: a call for a global research agenda. Lancet Psychiatry 11, 316–317. doi: 10.1016/S2215-0366(24)00098-1 38631783

[B150] O'Gorman (2024). “Revisiting ethics in the context of climate breakdown,” in Being a Therapist in a Time of Climate Breakdown, eds. J. Anderson, T., Staunton, J. O'Gorman, and C. Hickman (London: Routledge), 42–53. doi: 10.4324/9781003436096-8

[B151] O'Hare D. P. (2022). The Climate Crisis, Children, Young People and Educational Psychology. British Psychology Society Division of Educational and Child Psychology (BPS DECP). Available online at: https://cms.bps.org.uk/sites/default/files/2022-07/DECP%20Discussion%20Paper%20-%20Climate%20Crisis%2C%20Children%20and%20Young%20People.pdf (Accessed May 8, 2026).

[B152] Ojala M. (2012). Hope and climate change: the importance of hope for environmental engagement among young people. Environ. Educ. Res. 18, 625–642. doi: 10.1080/13504622.2011.637157

[B153] Oreskes N. Conway E. (2011). Merchants of Doubt: How a Handful of Scientists Obscured the Truth on Issues from Tobacco Smoke to Climate Change. New York, NY: Bloomsbury.

[B154] Organizing: People Power, Change. (n.d.). Available online at: https://commonslibrary.org/wp-content/uploads/Organizers_Handbook.pdf (Accessed May 8, 2026).

[B155] Ozden J. Glover S. (2022). Protest Movements: How Effective Are They? Social Change Lab. Available online at: https://commonslibrary.org/protest-movements-how-effective-are-they/ (Accessed May 8, 2026).

[B156] Papies E. K. Nielsen K. S. Soares V. A. (2025). Health psychology and climate change: time to address humanity's most existential crisis. Health Psychol. Rev. 19, 463–493. doi: 10.1080/17437199.2024.2309242 38320578

[B157] Paxton R. Jones R. (2020). ‘I took my turn on Friday to be arrested'. The Psychologist 33, 28–32. Available online at: https://www.bps.org.uk/about-psychologist

[B158] Pearson A. R. Schuldt J. P. Romero-Canyas R. (2016). Social climate science: a new vista for psychological science. Perspect. Psychol. Sci. 11, 632–650. doi: 10.1177/1745691616639726 27694459

[B159] Pelletier L. G. Sharp E. (2008). Persuasive communication and pro environmental behaviours: how message tailoring and message framing can improve the integration of behaviours through self-determined motivation. Can. Psychol. 49, 210–217. doi: 10.1037/a0012755

[B160] Perrin A. J. Capstick S. Elliott T. Knapp P. Thierry A. Wyatt T. D. . (2025). How scientists can contribute to the social movements essential to protecting climate and nature, NPJ Clim. Action 4:72. doi: 10.1038/s44168-025-00268-9 40756286 PMC12313518

[B161] Pichler P. Jaccard I. Weisz, U. Weisz H. (2019). International comparison of health care carbon footprints. Environ. Res. Lett. 14:14064004. doi: 10.1088/1748-9326/ab19e1

[B162] Pigott P. Nuuttila H. Thomas M. Smith F Bohata K. Murray T. . (2024). “No one talks about it”: using emotional methodologies to overcome climate silence and inertia in higher education. Front. Sociol. 9:1456393. doi: 10.3389/fsoc.2024.1456393 39664602 PMC11631935

[B163] Pihkala P. (2020). The cost of bearing witness to the environmental crisis: vicarious traumatization and dealing with secondary traumatic stress among environmental researchers. Soc. Epistemol. 34, 86–100. doi: 10.1080/02691728.2019.1681560

[B164] Pihkala P. (2022). The process of eco-anxiety and ecological grief: a narrative review and a new proposal. Sustainability 14:16628. doi: 10.3390/su142416628

[B165] Pihkala P. (2024). “Climate sorrow: discerning various forms of climate grief and responding to them as a therapist,” in Being a Therapist in a Time of Climate Breakdown, eds. J. Anderson, T. Staunton, J. O'Gorman, C. Hickman, C., and D. Orange (London: Routledge), 157–168. doi: 10.4324/9781003436096-24

[B166] Power E. McCarthy N. Cannon M. Cotter D. (2022). Climate change and mental health: time for action and advocacy. Ir. J. Psychol. Med. 40, 6–8. doi: 10.1017/ipm.2021.70 35067251

[B167] Racimo F. Valentini E. Rijo De León G Santos, T. L. Norberg A. Atmore L. M. Murray M. . (2022). The biospheric emergency calls for scientists to change tactics. Elife 7:e83292. doi: 10.7554/eLife.83292 PMC9640186 36342018

[B168] Richie C. (2022). Environmental sustainability and the carbon emissions of pharmaceuticals. J. Med. Ethics 48:334. doi: 10.1136/medethics-2020-106842 33853877

[B169] Rocque R. J. Beaudoin C. Ndjaboue R. Cameron L. Poirier-Bergeron L. Poulin-Rheault R.-A. . (2021). Health effects of climate change: an overview of systematic reviews. Br. Med. J. Open 11:e046333. doi: 10.1136/bmjopen-2020-046333 PMC8191619 34108165

[B170] Romanello M , Di Napoli, C. Drummond P. Green C. Kennard H. Lampard P. Scamman D. . (2022). The 2022 report of the Lancet Countdown on health and climate change: health at the mercy of fossil fuels. Lancet 400, 1619–1654. doi: 10.1016/S0140-6736(22)01540-9 36306815 PMC7616806

[B171] Romanello M. McGushin A. Di Napoli C. D. Drummond P. Hughes N. Jamart L. . (2021). The 2021 report of the Lancet Countdown on health and climate change: code Red for a healthy future. Lancet 398, 1619–1662. doi: 10.1016/S0140-6736(21)01787-6 34687662 PMC7616807

[B172] Rossa-Roccor V. Giang A. Kershaw P. (2021). Framing climate change as a human health issue: enough to tip the scale in climate policy? Lancet Planet Health 5, e553–e559. doi: 10.1016/S2542-5196(21)00113-3 34390673

[B173] Royal College of Psychiatrists (2021). Our Planet's Climate and Ecological Emergency. Position Statement. Available online at: https://www.rcpsych.ac.uk/docs/default-source/improving-care/better-mh-policy/position-statements/position-statement-ps03-21-climate-and-ecological-emergencies-2021 (Accessed May 8, 2026).

[B174] Saleem S. (2026). Zohran Mamdani and community organising: the campaign that billionaires couldn't buy. The News Minute. Available online at: https://www.thenewsminute.com/news/zohran-mamdani-and-community-organising-the-campaign-that-billionaires-couldnt-buy (Accessed May 8, 2026).

[B175] Simmons A. (2008). “Civil disobedience and the duty to obey the law,” in A Companion to Applied Ethics, eds. R. G. Frey, and C. H. Wellman (Chichester: John Wiley and Sons), 50–61. doi: 10.1002/9780470996621.ch4

[B176] Singh G. Xue S. Poukhovski-Sheremetyev F. (2022). Climate emergency, young people and mental health: time for justice and health professional action. BMJ Paediatr. Open 6:e001375. doi: 10.1136/bmjpo-2021-001375 PMC8981321 36053656

[B177] Solomon C. LaRocque R (2019). Climate change - a health emergency. N. Engl. J. Med. 380, 209–211. doi: 10.1056/NEJMp1817067 30650319

[B178] Stoddard I. Anderson K. Capstick S. Carton W. Depledge J. Facer K. . (2021). Three decades of climate mitigation: why haven't we bent the global emissions curve? Annu. Rev. Clim. Resour. 46, 653–689. doi: 10.1146/annurev-environ-012220-011104

[B179] Tannock S. (2024). Adaptation strategies: labour education, climate change and the trade union movement. Glob. Labour Mov. 15:111. doi: 10.15173/glj.v15i2.5576

[B180] Taylor G. (2023). Ontario health workers push pension fund to divest fossil fuels by 2025. The Energy Mix. Available online at: https://www.theenergymix.com/ontario-health-workers-push-pension-fund-to-divest-fossil-fuels-by-2025/ (Accessed May 8, 2026).

[B181] Tennison I. Roschnik S. Ashby, B. Boyd R. Hamilton I. Oreszczyn, T. . (2021). Health care's response to climate change: a carbon footprint assessment of the NHS in England. Lancet Planet. Health 5, e84–e92. doi: 10.1016/S2542-5196(20)30271-0 33581070 PMC7887664

[B182] The Lancet (2020). Doctors and civil disobedience. Lancet 395:248. doi: 10.1016/S0140-6736(20)30120-3 31982049

[B183] Thierry A. Horn L. von Hellermann P. Gardner C. J. (2023). ‘No research on a dead planet': preserving the socio-ecological conditions for academia. Front. Educ. 8:1237076. doi: 10.3389/feduc.2023.1237076

[B184] Thomas A. (2021). Framing the just transition: how international trade unions engage with UN climate negotiations. Glob. Environ. Change 70:102347. doi: 10.1016/j.gloenvcha.2021.102347

[B185] Thomas-Walters L. Scheuch E. G. Ong A. Goldberg M. H. (2025). The impacts of climate activism. Curr. Opin. Behav. Sci. 63:101498. doi: 10.1016/j.cobeha.2025.101498

[B186] Timmermann C. Pascale Wabnitz K. J. Schlögl-Flierl K. Wild V. (2026). Physicians' ethical responsibilities in relation to the climate and further environmental crises: a review of academic publications. Med. Health Care Philos. 29, 559–576. doi: 10.1007/s11019-026-10339-1 41796424 PMC13086663

[B187] Tindall D. B. Kay F. M. Zuberi D. M. Bates K. L. (2008). “Urban and community studies,” in Encyclopedia of Violence, Peace, and Conflict, 3rd Edn, ed. L. R. Kurtz (San Diego, CA: Academic Press), 188–205. doi: 10.1016/B978-0-12-820195-4.00261-2

[B188] UNESCO (2021). Getting Every School Climate Ready: How Countries Are Integrating Climate Change Issues in Education [report]. Available online at: https://www.unesco-floods.eu/wp-content/uploads/2021/12/379591eng.pdf (Accessed May 8, 2026).

[B189] UNICEF (2015). Unless We Act Now: The Impact on Climate Change on Children. Available online at: https://sdgs.un.org/sites/default/files/publications/2161unicef.pdf (Accessed May 8, 2026).

[B190] United Nations (2025). Climate emergency is a health crisis ‘that is already killing us' says WHO. UN News. Available online at: https://news.un.org/en/story/2025/06/1164261 (Accessed May 8, 2026).

[B191] Urai A. E. Kelly C. (2023). Point of view: rethinking academia in a time of climate crisis. eLife 12:e84991. doi: 10.7554/eLife.84991 36748915 PMC9904754

[B192] Valero M. V. (2025). Helping youth move from climate anxiety to climate action. Monit. Psychol. 56:46.

[B193] van der Linden S. L. Leiserowitz A. Feinberg G. D. Maibach E. W. (2015). The scientific consensus on climate change as a gateway belief: experimental evidence. PLoS One 10:e011848. doi: 10.1371/journal.pone.0118489 PMC4340922 25714347

[B194] Van Susteren L. (2018). The psychological impacts of the climate crisis: a call to action. Br. J. Psychol. Int. 15, 25–26. doi: 10.1192/bji.2017.40 PMC6020909 29953119

[B195] Vestergren S. Bamberg S. Louis W. (2024). Responding to the socio-ecological crisis: activism and collective action. Glob. Environ. Psychol. 2:e13075. doi: 10.5964/gep.13075

[B196] Vestergren S. Drury J. Chiriac E. H. (2018). How collective action produces psychological change and how that change endures over time: a case study of an environmental campaign. Br. J. Soc. Psychol. 57, 855–877. doi: 10.1111/bjso.12270 30079590 PMC6220852

[B197] Vogt J. Subasinghe R. (2025). Turning up the heat: the right to strike and the climate crisis. Comp. Lab. Law Policy J. 45:10. doi: 10.60082/2819-2567.1042

[B198] Waddington K. (2025). Launch of New National Campaign to Reduce Reuse Plastics in Health Care [media release]. Available online at: https://greenhealthcare.ca/launch-of-new-national-campaign-to-reduce-reuse-plastics-in-health-care/ (Accessed May 8, 2026).

[B199] Wallen K. Landen A. C. (2020). Systematic map of conservation psychology. Conserv. Biol. 34, 1339–1352. doi: 10.1111/cobi.13623 33245809 PMC7756398

[B200] Weintrobe S. (2021). Psychological Roots of the Climate Crisis: Neoliberal Exceptionalism and the Culture of Uncare. London: Bloomsbury. doi: 10.5040/9781501372902

[B201] Whitburn J. Linklater W. Abrahamse W. (2019). Meta-analysis of human connection to nature and pro environmental behaviour. Conserv. Biol. 34, 180–193. doi: 10.1111/cobi.13381 31251416 PMC7027494

[B202] Whitmarsh L. Capstick S. Moore I. Jana Köhler, J. Le Quéré C. (2020). Use of aviation by climate change researchers: structural influences, personal attitudes, and information provision. Glob. Environ. Change 65:102184. doi: 10.1016/j.gloenvcha.2020.102184

[B203] Whitmarsh L. (2020). Almost every area of psychology has something to contribute to addressing climate change. The Psychologist 33, 24–26. Available online at: https://www.bps.org.uk/about-psychologist

[B204] Wray B. (2022). Gen Dread: Finding Purpose in an Age of Climate Crisis. Toronto, ON: Penguin Random House Canada.

[B205] Wyatt T. Gardner C. J. Thierry A. (2024). Actions speak louder than words: the case for responsible scientific activism in an era of planetary emergency. R. Soc. Open Sci. 11:11240411. doi: 10.1098/rsos.240411 PMC11251756 39021783

[B206] Yassaie R. (2024). Nonviolent Climate Protests and the Medical Profession – Should Doctors be Struck Off for Their Actions? Journal of Medical Ethics: forum. Available online at: https://blogs.bmj.com/medical-ethics/2024/04/18/nonviolent-climate-protests-and-the-medical-profession-should-doctors-be-struck-off-for-their-action (Accessed May 8, 2026).

[B207] Yassaie R. Brooks L. (2025). Reassessing ‘good' medical practice and the climate crisis. Br. Med. J. 51, 365–370. doi: 10.1136/jme-2023-109713 38871401

[B208] Yeon P. S. Jeon J. Y. Jung M. S. Min G. M. Kim G. Y. Han K. M. . (2021). Effect of forest therapy on depression and anxiety: a systematic review and meta-analysis. Int. J. Environ. Res. Public Health 18:12685. doi: 10.3390/ijerph182312685 34886407 PMC8657257

[B209] Young K. A. Thomas-Walters L. (2024). What the climate movement's debate about disruption gets wrong. Humanit. Soc. Sci. Commun. 11, 1–7. doi: 10.1057/s41599-023-02507-y

[B210] Zhao J. Radke J. Chen F. S. Sachdeva S. Gershman S. J. Luo Y. (2024). How do we reinforce climate action? Sustain. Sci. 19, 1503–1517. doi: 10.1007/s11625-024-01486-6

