# Supplementary material for: Ethics and action pathways for psychologists in a time of climate crisis
Source: Front Psychol. 2026 May 21;17:1770041. doi: 10.3389/fpsyg.2026.1770041 (PMC13233231; doi:10.3389/fpsyg.2026.1770041)
Supplement: Supplementary file 1 [file Supplementary_File_1.docx]

**Supplement A**

**Case Examples of Ethical Dimensions of Actions/Advocacy**

| Strategy | Examples of tactics and ethical dimensions |
| --- | --- |
| Individual Action | **Example 1** In celebration of Earth Day, your workplace invites you to ‘do your part’ to reduce your carbon footprint (i.e. reduce carbon emissions). For example, you are asked to compost, use less plastic, cycle to work, and eat plant-based food. However, there is little system infrastructure to support these actions. There are almost no plant-based options in the cafeteria, only one compost location on site (in the basement), no bike lanes in the community or bike storage on site, poor public transport, cafeteria vendors use only plastic beverage containers. *Considering the physical and mental health consequences of pollution and the climate crisis, is there an ethical responsibility to engage in sustainable practices at work? If there is a high burden placed on the individual with a low payoff regarding efficacy of the action, how does this impact the ethical dimension of this action?*  **Example 2** You are a psychology student. You are in your late 20s and are very concerned about your ability to pay off student debts, save for retirement, and secure a downpayment for a house. Also, you are worried about your ability to compete for residency positions. You are aware of the short- and long-term urgency of the climate crisis and the differential impact on individuals living in marginalized communities and on younger generations. You are aware of the investment of your university and the national pension plan in fossil fuels. You are aware of the improved impact of collective action over individual actions. *Considering the consequences of pollution and the climate crisis, your relative vulnerability and also your commitment to ethical conduct, what is your ethical responsibility and scope for action?* |
|  |  |
| Individual Advocacy | **Example 1** You are a psychologist with recognition for research in your field. As an individual employee, you set up a meeting with a member of the senior leadership team of your institution to discuss improving sustainability practices within the organization (e.g., in procurement, in food services, waste management, and responsible pension divestment from fossil fuels). Within the meeting, the senior leadership team member mentions that they appreciate your contributions and are grateful that you are willing to be ‘reasonable’ in your advocacy for more sustainable practices - unlike other complaints they have received from vocal students/junior colleagues. They agree to bring up one of your requests (extra recycling bins) - but not the others (divesting pensions from fossil fuels) - at the AGM. *How do you respond to this concession? Is there an ethical responsibility to engage in this type of advocacy? What voices are potentially left out in this model of advocacy? Does pursuing this tactic instead of collective action delay more effective and necessary mass action?* |
| Community Collective Action | **Example 1:** You are a psychologist living in a remote community, and evidence emerges that a mill is emitting effluent beyond the legal limit. The company pays the fine (which barely impacts their profit margin) and continues to emit. On culturally loaded measures, you are also aware that the community impacted by the effluent emission has a much higher rate of learning disabilities and other cognitive difficulties than national averages. Research indicates that exposure to elevated emissions is linked to cognitive deficits. In solidarity with the community, you have written letters and advocated many times to local and regional politicians regarding this issue. Nothing has changed. In desperation, community members organize to block factory workers from entering the mill in an attempt to garner media attention and pressure politicians and decision makers to take this case seriously and inhibit the factory from emitting. Community members have few financial resources and will likely be arrested and face legal consequences for this action. *What factors do you consider when weighing the decision to participate in blocking the factory (i.e. participate in nonviolent civil disobedience) or not? Do you participate (or not) as a community member? Should you participate as a health professional, even if you risk arrest, to demonstrate to politicians and the public that there is legitimate cause for concern and knowing your social capital will lend credibility to the action? Alternatively, to protect the reputation of psychology and because the relationship between effluent and disorders is not clearly causal, do you stand back and be available to provide emotional support to participants and their family members and support in other ways (e.g., to be an expert commentator to the media)?*  **Example 2:** You are a psychologist (or psychology student).  You work in a building in a rural community with 10 other small businesses including other health-related private practices. The local hospital in your community experiences frequent emergency room (ER) closures due to provincial funding cuts. There is a federal election approaching, and a well-known community leader is running, based on a platform of small increases in healthcare funding and for a political party that generally advocates for climate action. You are invited to work with an existing climate and health advocacy group to circulate an ‘open letter’ to every business in your building in order to 1. Push the candidate to commit to advocate for federal funding to ensure ER closures are eliminated and to 2. Sign on to the ‘Fossil Fuel Non-Proliferation Treaty’. You are asked to ‘sign on’ as a registered psychologist (or student) and to meet with the candidate with a group of community healthcare workers to deliver the petition. *How do you respond? How do you balance the critical need for political action to prevent further climate degradation with your social capital and reputation as a psychologist? Are there any implications in taking part in delivering this petition and thus acting as a public figure (even if in a limited capacity)?*  **Example 2 Cont.** The candidate commits to these demands, and you participate in the electoral campaign (as a private citizen, although known as a psychologist) to mobilize voters to support this candidate (e.g., by encouraging volunteers to engage in ‘deep canvassing’ with potential voters, a tactic with evidence for effectiveness). However, the media focused on other issues. One month before the election, you are invited to a rally at the federal parliament to help bring publicity to climate demands. Also, you are asked to organize a bus contingent to bring members of your community to the rally. You are given a ‘heads up’ that some members of this action will be participating in a one hour ‘sit-in’ (i.e. an act of nonviolent civil disobedience) in front of parliament, during rush hour. *What level of participation do you take in the rally? Do you lend your ‘social capital’ as a contingent of health workers? How do psychologists weigh the relative risk of inaction on climate and the possible risk associated with participation in non-violent civil disobedience with the reputation of psychology?* |
| Worker Collective Action | **Example 1** You are a psychologist (psychology student) who works on the eating disorder unit at a public hospital in a major city. Due to funding cuts, there is a staffing crisis at the hospital, and many health workers have quit due to burnout. Last summer, there was a ‘code orange’ as a result of a massive nearby forest fire. Additionally, there have been two major ‘heat dome’ events which caused an increase in ER admissions and several hundred premature deaths in the city. During these events, and at other times during the year, there is increased pressure on all staff to be reassigned, to cover more clients (patients) than is safe, and to perform duties outside of their scope of practice. You regularly attend union meetings. However, few other psychologists do so. An ‘open letter’ to demand ‘set staffing ratios’ and to ensure that these ratios can be maintained during increasingly common unnatural weather events is generated. The letter also demands that the health organization develops a ‘climate adaptation’ plan to ensure that the health centre buildings have up-to-date cooling systems that can withstand heat waves and that the health organization sign on to support the ‘Leap Manifesto’. As per established models for collective action organizing (c.f. McAlevey, 2016), you and an influential nurse, map out your unit, schedule one-on-one conversations with health workers. As a result, 90% of your colleagues sign the open letter. This letter is delivered to the head of the hospital and to your province's premier. *Would you consider engaging clients/patients in conversations about broader health/climate systems change as it pertains to their health and level of care? Is there a greater responsibility to act as a worker in a secure, public, unionized position within healthcare spaces?*  **Example 1 cont.** Lobbyists for the fossil fuel industry have been granted access to meet with elected officials many times more frequently than environmental/climate organizations as well as funding public disinformation campaigns. Your letter is ignored. In an effort to increase political pressure on decision makers, you are asked by a trusted colleague to help organize a highly publicized ‘work to rule’ action, where workers do not perform duties outside of their scope of practice or accept unsafe health practitioner: client/patient ratios for a week. To gain media attention, you are invited to join a client-practitioner advocacy group to host a ‘die-in’ rally in front of city hall. Protesters hold signs that say, “Worker health = client health = climate health”. The city council votes to sign onto the ‘Leap Manifesto’ and advocate to the government for increased hospital funding. *Considering the ‘work-to-rule’ action, how do workers weigh the short, medium and long-term risks of working in unsafe client/patient: staff ratios with the short-term risks to access to care for clients?* |
