# Supplementary material for: Ethics and action pathways for psychologists in a time of climate crisis
Source: Front Psychol. 2026 May 21;17:1770041. doi: 10.3389/fpsyg.2026.1770041 (PMC13233231; doi:10.3389/fpsyg.2026.1770041)
Supplement: Supplementary file 2 [file Supplementary_File_2.docx]

**Supplement B**

Supplementary Table 1

*Relevant Health and Academic Organizations Involved in Climate/ Environmental Advocacy and Activism*

| Organisation | Purpose | Focus |
| --- | --- | --- |
| CASCADES  https://cascadescanada.ca | Supports the creation of a sustainable healthcare system in a climate crisis | Educational resources for healthcare facilities.  Training for the sustainability journey of healthcare.  Connection with other healthcare facilities creating change. |
| Canadian Association of Physicians for the Environment  https://cape.ca/ | Open to all, including psychologists.  Advocates for mitigation, environmental health and sustainability | Mitigation focused. Advocacy to elected officials. Campaigns related to health/ climate (e.g. banning fossil fuel advertising; health and economic policy; preventing toxic exposures) |
| Canadian Coalition for Green Health Care  https://greenhealthcare.ca/ | Supports sustainable health service delivery systems | Resources, for example related to health care forests, environmental stewardship, GHG and water, Green Hospital Scorecard, Sustainable prescribing, procurement, menus, |
| Canadian Health Association for Sustainability and Equity  https://chasecanada.org/ | Works towards sustainable health systems and equity in health. | Research on health equity and sustainability, Develops policy recommendations |
| Canadian Coalition for Green Healthcare<https://greenhealthcare.ca/> | Aims to reduce the environmental impact of healthcare in Canada. | Waste reduction energy efficiency in healthcare facilities, Collaboration across agencies, education |
| Climate Minds Coalition  https://www.climatemindscoalition.com/ | Aims to work with health services and political leaders to build and improve access to high quality support for people experiencing the psychological impacts of climate change. | Training and support for mental health professionals working with those negatively impacted by climate change  Support efforts to protect the environment  Support and collaboration for mental health professionals dealing with emotional reactions to climate change  Advocacy to increase access of public to options to increase coping  Work with political leaders to understand reactions to the climate emergency and what can be done to help |
| Climate Psychiatry Alliance [www.climatepsychiatryalliance.org](http://www.climatepsychiatryalliance.org/) | Focuses on the mental health impacts of climate change. | Climate anxiety research.  Resources for mental health professionals. |
| Climate Psychology Alliance  [www.climatepsychologyalliance.org](http://www.climatepsychologyalliance.org/) | Enhances the understanding of the psychological impacts of climate change | Adaptation focused i.e. processing psychological responses to the climate crisis; building resilience.  Education, training for psychologists related to responding to psychological impacts of climate change. |
| Climate Social Science Network  https://cssn.org/ | Promotion of research focused on understanding political conflict over climate change | Facilitate international network of climate scholars, host events, provide grants to prompt research for social scientists |
| Ecopsychepedia  https://ecopsychepedia.org/ | Source of information on how human activity has caused the climate crisis, the impacts of the crisis and what can be done about it. | Resources for mental health professionals, educators, parents and caregivers |
| Evidence for Democracy  https://evidencefordemocracy.ca | Putting evidence at the heart of public policy in Canada | Informing public policy with science; presenting talks, panels, webinars, training, marches, evidence-based research reports |
| Global Climate and Health Alliance [www.climateandhealthalliance.org](http://www.climateandhealthalliance.org/) | Promotes global collaboration on health and climate initiatives. | International summits, collaborative projects addressing climate. Emphasizes partnerships and global dialogue |
| Health for XR  [www.healthforxr.org](http://www.healthforxr.org/)  (UK based) | Mobilizes healthcare professionals to advocate for climate action. | Organized campaigns focused on mitigation, raising awareness. Public peaceful protest, including NVCD |
| HUB  https://www.lehub.ca/en/ | Free training events and resources relevant for collective action organizing | Mitigation focused. Hosts training webinars and provides resources. |
| Mental Health Climate Change Alliance  https://mhcca.ca/ | Helping to navigate emotional responses to climate change | Adaptation focused i.e. navigating feelings about climate. Research, conference. |
| PEACH Health Ontario [www.peachhealthontario.ca](http://www.peachhealthontario.ca/) | Promotes environmental health and sustainability in health care organizations. | Advocacy, community engagement, webinars, toolkits, case studies’ of sustainable innovation in healthcare |
| [Planetary Health Organizations for Wellbeing, Equity & Regeneration](https://www.linkedin.com/company/power-wellbeing/)  https://www.powerwellbeing.ca/ | Pan-Canadian network dedicated to wellbeing, equity, and regeneration for future generations | Building a pan-Canadian network of “Think and Change” nodes which bridge academia, healthcare, and the community. They are linked together by a national secretariat to deliver the coordination, knowledge mobilization, and strategic, evidence-based communications required to adapt our society and economy with planetary health and wellbeing principles. |
| Psychology Coalitions of NGOs at the United Nations  The Psychology Coalition at the United Nations (PCUN)  https://psychologycoalitionun.org/ | The Psychology Coalition at the United Nations (PCUN) is composed of representatives of psychology and psychology-related organizations that are non-governmental organizations (NGOs) with consultative status to the United Nations (UN) Economic and Social Council (ECOSOC), and those affiliated with UN departments, agencies and missions. | Webinars, papers to offer timely, objective, and data-based information to the United Nations and other key stakeholders about current and emerging issues facing diverse communities. Harnesses expertise from psychology to identify and promote promising practices and actionable steps in response to global concerns. |
| Scientists for Global Responsibility  <http://www.sgr.org.uk/> | Promoting ethical science and technology | Mitigation, collective action to promote social justice. Webinars/ resources for (e.g. fair lifestyle targets, nuclear weapons threat, corporate influence on science and technology) |
| Social Change Lab  https://www.socialchangelab.org/ | Researches social movements to understand their impact. | Evidence synthesis for social movement, activist resources, tactic sheets, research summaries |
| The Commons: Social Change Library  <https://commonslibrary.org/> | Resources to support novice and experienced advocates and activists | 1500+ educational accessible free resources in a range of formats. Topics include campaign strategy, community organizing, working effectively in groups, justice and diversity, creative activism. |
| The Sunrise Movement  https://www.sunrisemovement.org/latest/#resources | Sunrise is building a movement of young people to end billionaire rule and stop the climate crisis. | Grassroots organizing in youth. Toolkits and resources. |
| Yale Program on Climate Communications  https://climatecommunication.yale.edu/ | Research on public climate change knowledge, attitudes, policy preferences, and behavior, and the underlying factors that influence them. | Resources relevant to evidence synthesis, webinars, messaging, specific audiences, policy |

*Note.* The table contains a list of some academic and psychology-related organizations involved in environmental advocacy and activism. The list is not exhaustive. This list is not considered a specific endorsement by the authors who encourage readers to evaluate them, their focus and the degree to which they align with their values.
